# Asymmetric Hapln1a drives regionalized cardiac ECM expansion and promotes heart morphogenesis in zebrafish development

**DOI:** 10.1093/cvr/cvab004

**Published:** 2021-02-22

**Authors:** Christopher J Derrick, Juliana Sánchez-Posada, Farah Hussein, Federico Tessadori, Eric J G Pollitt, Aaron M Savage, Robert N Wilkinson, Timothy J Chico, Fredericus J van Eeden, Jeroen Bakkers, Emily S Noël

**Affiliations:** 1 Department of Biomedical Science, University of Sheffield, Western Bank, Sheffield, S10 2TN, UK; 2 Hubrecht Institute for Developmental and Stem Cell Biology, Uppsalalaan 8, 3584 CT, Utrecht, Netherlands; 3 Department of Infection, Immunity and Cardiovascular Disease, University of Sheffield, Medical School, Beech Hill Road, Sheffield, S10 2RX, UK

**Keywords:** Heart morphogenesis, Extracellular matrix, Laterality, Zebrafish, Heart development

## Abstract

**Aims:**

Vertebrate heart development requires the complex morphogenesis of a linear tube to form the mature organ, a process essential for correct cardiac form and function, requiring coordination of embryonic laterality, cardiac growth, and regionalized cellular changes. While previous studies have demonstrated broad requirements for extracellular matrix (ECM) components in cardiac morphogenesis, we hypothesized that ECM regionalization may fine tune cardiac shape during heart development.

**Methods and results:**

Using live *in vivo* light sheet imaging of zebrafish embryos, we describe a left-sided expansion of the ECM between the myocardium and endocardium prior to the onset of heart looping and chamber ballooning. Analysis using an ECM sensor revealed the cardiac ECM is further regionalized along the atrioventricular axis. Spatial transcriptomic analysis of gene expression in the heart tube identified candidate genes that may drive ECM expansion. This approach identified regionalized expression of *hapln1a,* encoding an ECM cross-linking protein. Validation of transcriptomic data by *in situ* hybridization confirmed regionalized *hapln1a* expression in the heart, with highest levels of expression in the future atrium and on the left side of the tube, overlapping with the observed ECM expansion. Analysis of CRISPR-Cas9-generated *hapln1a* mutants revealed a reduction in atrial size and reduced chamber ballooning. Loss-of-function analysis demonstrated that ECM expansion is dependent upon Hapln1a, together supporting a role for Hapln1a in regionalized ECM modulation and cardiac morphogenesis. Analysis of *hapln1a* expression in zebrafish mutants with randomized or absent embryonic left–right asymmetry revealed that laterality cues position *hapln1a*-expressing cells asymmetrically in the left side of the heart tube.

**Conclusion:**

We identify a regionalized ECM expansion in the heart tube which promotes correct heart development, and propose a novel model whereby embryonic laterality cues orient the axis of ECM asymmetry in the heart, suggesting these two pathways interact to promote robust cardiac morphogenesis.

## 1. Introduction

Congenital heart defects are the most common human birth abnormality, with an incidence of approximately 1% of live births.^1^ These structural malformations arise due to abnormal morphogenesis and maturation of the heart during embryonic development. A key stage in cardiac development occurs when the heart transitions from a linear tube to an asymmetric organ, a process including initial looping morphogenesis of the tube and subsequent ballooning of the cardiac chambers. Correct cardiac morphogenesis is vital for ensuring normal blood flow through the heart, proper chamber and vessel alignment, valve formation and septation, and is therefore a tightly controlled process requiring intricate coordination of heart-extrinsic signalling cues, cardiac growth, and tissue-intrinsic changes in cell shape.^2^

The requirement for embryonic left–right signalling pathways in promoting directionality of heart morphogenesis is well established, with asymmetric Nodal signalling playing a key role in driving rightward looping of the linear heart tube in multiple organisms.^3–6^ However, while embryos with defective asymmetric Nodal signalling display disrupted directionality of heart looping, the heart still undergoes looping morphogenesis.^5–7^ This indicates that while extrinsic asymmetric cues provide directional information to the heart, regionalized intrinsic signals may help to promote morphogenesis. How the interplay of both extrinsic and intrinsic regionalized signalling and cell behaviours ensures the coordination of directionality and morphogenesis required to orient and shape the heart remains unknown.

The developing heart tube is composed of two tissue layers: an outer tube of myocardium surrounding an inner layer of specialized endothelial cells (endocardium). These two layers are separated by an extracellular matrix (ECM), termed the cardiac jelly. The ECM consists of collagens, glycosaminoglycans, and glycoproteins and plays a pivotal role in providing mechanical cues and modulating extracellular signalling in the heart during cardiac development.^8^ Classic embryological experiments demonstrated that the cardiac jelly is important for heart morphogenesis^9^ while more recent studies have begun to identify specific ECM constituents with distinct roles in heart development.^10–16^ Hyaluronic acid (HA) is a glycosaminoglycan with conserved roles in heart tube formation, cardiac morphogenesis and atrioventricular valve development,^10^^,^^17^^,^^18^ suggesting multiple requirements for HA at various stages during cardiac development. While broad disruption of the cardiac ECM has profound effects on heart morphology,^17^^,^^19^ it is likely that the ECM plays distinct functions in regulating regionalized morphogenesis of the heart tube.

In this study, we demonstrate that the cardiac ECM of the zebrafish heart tube exhibits regionalized expansion prior to onset of heart tube morphogenesis, with a thicker ECM in both the left side and future atrium of the heart tube. Loss-of-function analyses demonstrate that this ECM expansion is dependent upon the ECM cross-linking protein Hyaluronan and Proteoglycan Link Protein 1a (Hapln1a), and that Hapln1a promotes heart morphogenesis. Finally, we show that while asymmetric *hapln1a* expression is independent of laterality cues, the axis of *hapln1a* asymmetry in the heart is dictated by embryonic laterality, supporting a new model where embryonic left–right asymmetry tightly defines the orientation of ECM asymmetry in the heart tube, and together these pathways fine tune asymmetric cardiac morphogenesis.

## 2. Methods

### 2.1 Zebrafish maintenance

Adult zebrafish were maintained according to standard laboratory conditions. The following lines were used: AB, *Tg(myl7:eGFP)*,^20^  *Tg(myl7:lifeActGFP)*,^21^  *Tg(fli1a:AC-TagRFP),^sh511^*  ^22^  *spaw^t30973^ ^7^, Tg(lft2BAC:Gal4FF); Tg(UAS;RFP), pkd2^hu2173^, hapln1a^Δ187^* (allele designation *hapln1a^sh611^*), *hapln1a^Δ241^* (allele designation *hapln1a^sh580^*). Embryos older than 24hpf were treated with 0.2 mM 1-phenyl-2-thiourea (PTU) in E3 medium to inhibit melanin production. All animals were euthanized by immersion in overdose of Tricaine methanesulfonate (1.33 g/L). Animal work was approved by the local Animal Welfare and Ethical Review Body (AWERB) at the University of Sheffield, conducted in accordance with UK Home Office Regulations under PPLs 70/8588 and PA1C7120E, and in line with the guidelines from Directive 2010/63/EU of the European Parliament on the protection of animals used for scientific purposes.

### 2.2 Generation of *hapln1a* mutants

CRISPR guide RNAs (gRNAs) were designed to target the putative promoter region of *hapln1a* (GRCz11: ENSDART00000122966.4, g1: 5′-TCGTCTCTCTCTAAGGGGAGGGG-3′) and downstream of the translation start site (g2: 5′-GATGATTGCTCTGTTTTCTGTGG-3′). Sequence-specific CRISPR RNAs (crRNA, Merck) were resuspended in MilliQ water to 21.4 μM, and injected with equimolar tracrRNA (Merck) together with Cas9 protein (NEB M0386T) into 1-cell stage embryos in a volume of 1nl. CRISPR-Cas9-injected embryos were raised to adulthood and individuals transmitting germline promoter deletions were identified by PCR using the following primers: forward 5′-ACATTTTGCATGCCCTCGAA-3′; reverse 5′-TGCATCCTGGACCTTCATTCA-3′. Promoter deletions were identified by Sanger sequencing. F0 founders transmitting a desirable mutation were established as stable lines at F2. Two *hapln1a* promoter deletion alleles were recovered: *hapln1a^Δ187^* and *hapln1a^Δ241^*.

### 2.3 mRNA *in situ* hybridization

Embryos were fixed overnight in 4% PFA, and mRNA *in situ* hybridizations carried out as previously described.^7^ Fluorescent *in situ* hybridizations were performed using the TSA kit (Perkin-Elmer).^23^ Primers used to generate new mRNA *in situ* probe constructs and information on published probes are detailed in Supplementary material online, *Methods*. Riboprobes were transcribed from linearized template in the presence of DIG-11-UTP or Fluorescein-11-UTP (Roche).

### 2.4 Immunohistochemistry

Embryos were fixed overnight in 4% PFA with 4% sucrose at 4°C. For Hapln1a immunostaining samples were stored overnight in MeOH at −20°C. After rehydration if applicable, embryos were blocked for 1 h at room temperature in 0.2% PBS-Triton-X (PBS-Tx) with 10% goat serum. Embryos were incubated overnight at 4°C with primary and secondary antibodies diluted in PBS-Tx/10% goat serum with 1% DMSO. The following commercially available primary antibodies were used: αGFP (1:1000 Aves lab), αCT3 (1:100, Developmental Studies Hybridoma Bank), αCdh5^24^ (1:100). A rabbit polyclonal antibody targeting amino acids 117–134 (DGMNDMTLEVDLEVQGKD) of zebrafish Hapln1a was designed and produced by Proteintech. Test bleeds were used to determine cross-reactivity with Hapln1a by comparing protein localization at 26hpf with mRNA *in situ* hybridization. Subsequently, affinity-purified Hapln1a antibody was used at 1:100. Fluorophore-conjugated secondary antibodies (Jackson labs) were used at 1:200.

### 2.5 Tomo-seq

Hearts were dissected from *Tg(myl7: eGFP)* zebrafish embryos at 26hpf and placed into OCT cryofreezing medium (Sakura Finetek). Blue Affy-gel beads (BioRad) were placed at each end of the heart tube to aid visualization during sectioning, and the hearts were rapidly frozen and stored at −80°C. Hearts were sectioned using a cryostat at 9 nm resolution. RNA extraction, aRNA synthesis, library preparation, sequencing, and data analysis were performed as previously described.^25^^,^^26^

### 2.6 Morpholino-mediated knockdown and *hapln1a* overexpression construct generation and analysis

A morpholino was designed to target the translational start site of *hapln1a* (5′-AGAGCAAT[CAT]CTTCACGTTTGTTA-3′, brackets denote *hapln1a* ATG reverse complement). Morpholinos blocking *tp53*,^27^ (Zfin *tp53* MO-4) and *has2*^28^ (Zfin *has2* MO-1) are previously described. All morpholinos were supplied by GeneTools and diluted to a 1 mM stock. Working concentrations were as follows: *hapln1a* 500 nM or 250 nM, *has2* 250 nM, combinatorial *has2/hapln1a* 250 nM each, *tp53* 250 nM. *has2* and *hapln1a* morpholinos were co-injected together with *tp53* morpholino. Embryos were injected with 1 nL of morpholino solution.

The *hapln1a* coding sequence was amplified using the following primers containing AttB sequences for Gateway cloning and a Kozak sequence (underlined): forward: 5′ggggacaagtttgtacaaaaaagcaggctTCGCCGCCACCATGATTGCTCTGTTTTCTGT 3′; reverse: 5′GGGGACCACTTTGTACAAGAAAGCTGGGTTTTACTGCTGGGCTTTGTAGCAATA-3′. The resulting PCR product was ligated into the pDONR221 middle entry Gateway vector, generating a pME*hapln1a*CDS vector. Full-length *hapln1a* was subsequently recombined with a p5E *myl7* promoter sequence,^29^ and a p3E polyA sequence into the pDestTol2pA3 destination vector^30^ to generate the pDest*myl7*: *hapln1a* construct. Gateway cloning was performed using the Tol2kit via standard protocols.^30^ 60 pg of pDest*myl7: hapln1a* was co-injected with 25 pg of *tol2* mRNA into the cell of 1-cell stage embryos. Analysis of *hapln1a* overexpression and cardiac morphogenesis was performed using double *in situ* hybridization to assess *hapln1a* and *myl7* expression.

### 2.7 RNA injections

*ssNcan-GFP* mRNA was synthesized from the *ssNcan-GFP* plasmid as previously described.^31^ Embryos were injected with 100 pg of mRNA in 1 nL volume at the 1-cell stage and screened for GFP at 24hpf.

### 2.8 Imaging and image quantification

Live zebrafish embryos were imaged on a ZEISS Lightsheet Z.1 microscope. To assess cardiac morphology at 50hpf and 72hpf embryos were anesthetized by immersion in 8.4% Tricaine (Merck 10521) before mounting in 1% low melting point agarose in E3 with 8.4% Tricaine. To stop the heart the imaging chamber was filled with E3 with 8.4% Tricaine and the temperature maintained at 10°C. All samples were imaged using a 20× lens and 1.0 zoom at 0.47–0.65 µm z-step size, with sufficient z slices to capture the entire heart. Dual side lasers with dual side fusion and pivot scan were used for sample illumination.

Embryos injected with *ssNcan-GFP* mRNA were fixed overnight in 4% PFA with 4% sucrose, and the GFP signal amplified by immunohistochemistry. Dissected embryos were imaged using a Zeiss Airyscan microscope, z stacks were obtained with a step size of 1 µm.

Detailed image quantification methodology is included in Supplementary material online.

## 3. Results

### 3.1 The cardiac ECM is asymmetrically expanded at early stages of heart looping morphogenesis

During cardiac development the myocardial and endocardial layers of the heart are separated by the cardiac ECM. We hypothesized there may be regional differences in the ECM of the zebrafish heart tube which could promote local changes in tissue shape to drive cardiac morphogenesis. To examine regional ECM thickness in the heart tube, we used live *in vivo* light-sheet microscopy to image quadruple transgenic zebrafish embryos at 26 h post-fertilization (hpf).

**Figure 1:**
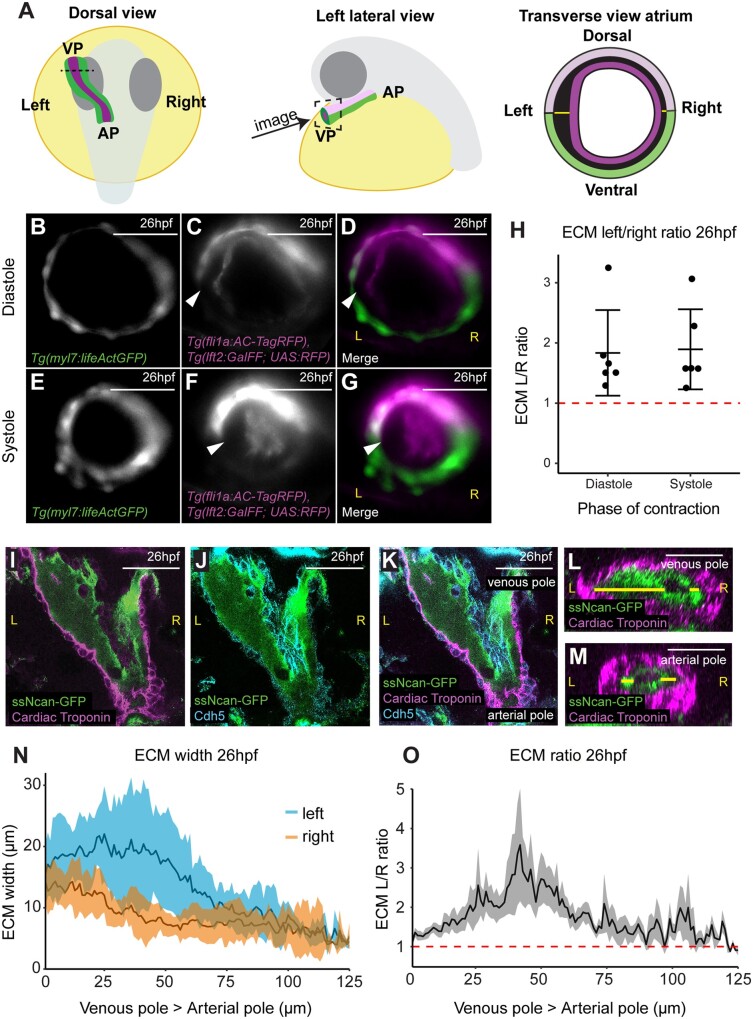
The hyaluronan-rich ECM is asymmetric during early zebrafish heart development. (*A*) Schematic depicting the developmental stage and orientation of zebrafish embryos used in live imaging experiments. Optical transverse sections of the heart tube are imaged at the position of the dotted line/dotted square. Green, myocardium; magenta, endocardium; light pink, dorsal myocardium. (*B–G*) Light-sheet optical cross-sections through the heart tube of a 26hpf *Tg(myl7:lifeActGFP); Tg(fli1a:AC-TagRFP); Tg(lft2BAC:Gal4FF); Tg(UAS:RFP)* transgenic embryo during diastole (*B–D*) and systole (*E–G*) at the level of the dotted line in (*A*). The myocardium is marked in green (*B, D, E*, and *G*), and the dorsal myocardium and endocardium are marked in magenta (*C, D, F*, and *G*). The extracellular space between myocardium and endocardium is expanded on the left side of the heart tube (white arrowhead). Scale bar = 50 μm. (*H*) Quantification of left–right ECM ratio in heart tubes, >1 (red dotted line) denotes left-sided expansion. Mean ± SD are plotted, *n* = 6. (*I–K*) Single confocal *z*-planes longitudinally through the heart at 26hpf of embryos injected with *ssNcan-GFP* (green), counterstained with cardiac troponin (magenta, *I, K*) and VE-Cadherin (cyan *J, K*). (*L* and *M*) Transverse optical reslice through the heart tube the venous pole (*L*) or arterial pole (*M*). ECM width is measured using the ssNcan-GFP signal (yellow line) on left and right sides of the tube. (*N*) Quantification of ECM width on the left (blue) and right (orange) sides of the heart tube from venous pole to arterial pole at 26hpf. Mean ± SD are plotted, *n* = 7. (*O*) Left–right ECM ratio in the heart tube from venous pole to arterial pole, where >1 (red dotted line) indicates a left-sided expansion. Mean ± SEM are plotted, *n* = 7. The mean L/R ratio across the heart is 1.667, and analysis using a one-sample *t*-test shows this significantly differs from 1, *P* < 0.0001 L, left; R, right; VP, venous pole; AP, arterial pole. Scale bar = 50μm.

*Tg(myl7:lifeActGFP); Tg(fli1a:AC-TagRFP); Tg(lft2BAC:Gal4FF); Tg(UAS:RFP)* zebrafish express actin-tagged GFP in the myocardium^21^ and actin-localized RFP in the endothelium including the endocardium,^22^ allowing visualization of the two tissue layers in the heart tube. The *Tg(lft2BAC:Gal4FF); Tg(UAS:RFP)* double transgenic drives RFP in *lefty2*-expressing cells, comprising the dorsal myocardium of the heart tube at 26hpf (*Figure 1A* and Supplementary material online, *Figure S1*).^18,32^ This combination of transgenes allowed imaging of optical cross-sections through the heart tube at 26hpf, just before the onset of looping morphogenesis, and enabled dorsal–ventral axis orientation of the heart tube (*Figure 1A–G* and Supplementary material online, *Movie S1*). We consistently observed an asymmetry in the extracellular space between the myocardial and endocardial layers of the heart in the future atrium, with an apparent thickening of the ECM on the left side of the tube which is maintained throughout the cardiac cycle (*Figure 1D and G*). We quantified the extracellular space between the two tissue layers and calculated the ECM left: right ratio (left ECM thickness divided by right ECM thickness, where >1 indicates a left sided expansion). Using this method, we detected a reproducible expansion of the ECM in the left side of the heart tube (*Figure 1H*) which is maintained in the atrium at 50hpf (Supplementary material online, *Figure S2*).

Due to technical limitations in imaging deeper cardiac tissue with sufficient resolution at 26hpf, we could only image the superficially located venous pole/atrium of the heart tube in live embryos. Therefore, to determine whether ECM left–right asymmetry is restricted to the venous pole or is maintained along the atrioventricular axis of the heart, we performed fixed tissue imaging. Previous studies have demonstrated that hyaluronic acid (HA) is present in the cardiac jelly during vertebrate heart development.^17^^,^^33^^,^^34^ To visualize the HA-rich ECM, wild-type embryos were injected with the HA sensor *ssNcan-GFP^31^* at the 1-cell stage, fixed at 26hpf, and the GFP signal detected by immunohistochemistry before imaging the entire heart tube as a z-stack using confocal microscopy (*Figure 1I–K*). Optical reslicing of z-stacks generated cross-sections of the heart tube from the venous pole to the arterial pole, allowing us to quantify the width of the *ssNcan-GFP*-positive ECM on left and right sides of the tube along the entire pole-to-pole length of the heart (*Figure 1L–N*). We confirmed that the ECM is thicker on the left side of the heart tube compared to the right, however this asymmetry is more profound at the venous pole/future atrium than at the arterial pole/future ventricle (*Figure 1N and O*). Furthermore, the cardiac ECM is thicker in the future atrium when compared to the ventricle (*Figure 1L–N*). Together these data demonstrate that the heart tube exhibits a regionally expanded ECM prior to onset of looping morphogenesis.

### 3.2 h*apln1a* exhibits regionalized cardiac expression prior to heart tube formation and looping morphogenesis

The asymmetric expansion of the cardiac ECM could be due to regionalized synthesis of ECM components. However, we did not observe any clear asymmetry in levels of HA deposition in the cardiac ECM in either live embryos injected with the *ssNcan-GFP* sensor (Supplementary material online, *Figure S3*) or in fixed hearts (*Figure 1I–K*). We also did not find left–right asymmetry in the heart tube in the expression of *hyaluronan synthase 2 (has2,* the major HA producing enzyme), *chondroitin sulfate synthase 1* (*chsy1*), or the ECM proteoglycans *versican* (*vcana/b), aggrecan (acana/b)*, all of which have previously been implicated in heart development^11^^,^^17–19^^,^^35^^,^^36^ (Supplementary material online, *Figure S3*), suggesting that regionalized synthesis of these proteins is not responsible for ECM asymmetry. We therefore hypothesized that a protein required for HA modification or cross-linking may be regionally expressed in the heart tube.

**Figure 2:**
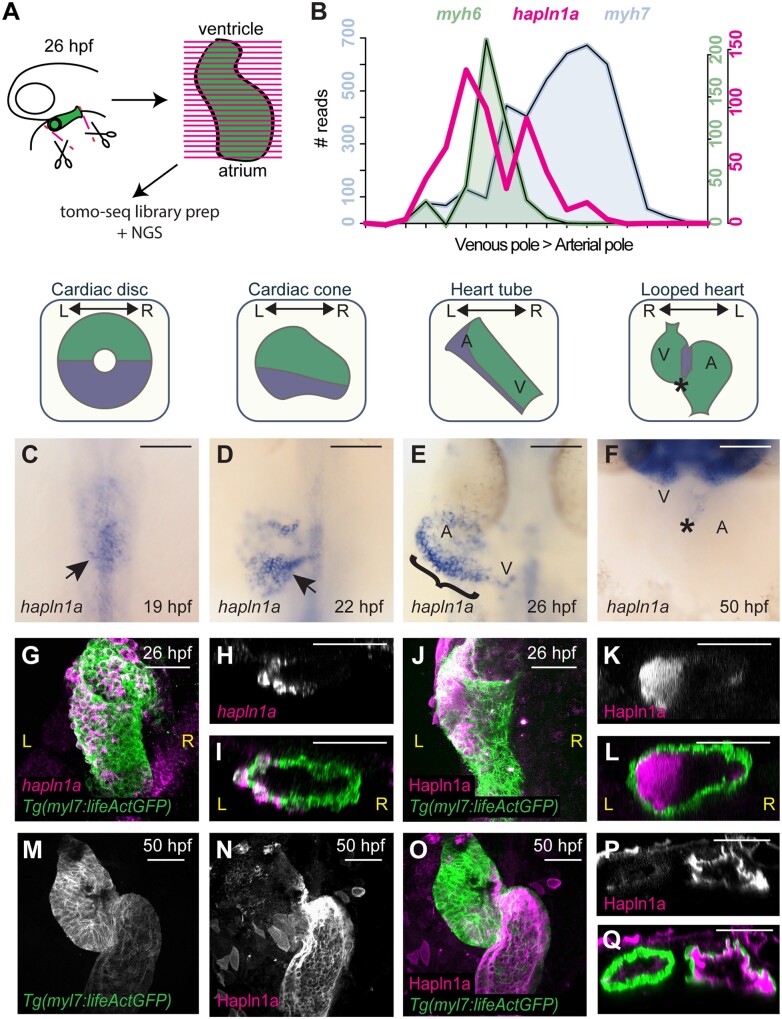
*hapln1a* is regionally expressed in the heart tube and secreted asymmetrically into the cardiac jelly. (*A*) Schematic representation of Tomo-seq pipeline. GFP-expressing hearts are manually excised from embryos at 26hpf, and frozen in tissue freezing medium. Heart tubes are sectioned along the atrioventricular axis. RNA extracted from individual slices is labelled with a slice-specific molecular barcode during reverse transcription before generating sequencing libraries. (*B*) Example Tomo-seq traces from a single 26hpf heart tube, with individual slices from venous pole to arterial pole along the *x*-axis and normalized read number along the *y*-axis. Read numbers for atrial marker *myh6* (green) and ventricular marker *myh7* (blue) allows identification of chamber position within the dataset. *hapln1a* expression (magenta) is up-regulated in atrial sections. (*C–F*) mRNA *in situ* hybridization analysis of *hapln1a* expression in the heart between 19hpf and 50hpf. At cardiac disc stage, *hapln1a* is up-regulated in the posterior (arrow *C*), which is maintained as the heart forms the cardiac cone prior to tube formation (arrow *D*), with lower expression in the anterior cone. Once the heart cone has extended to form the tube, the previously posterior *hapln1a* expression is positioned on the left side of the tube (bracket, *E*), and expressed at higher levels in the atrium (A) than the ventricle (V). By 50hpf *hapln1a* expression in the heart is restricted to low levels in the atrioventricular canal (AVC, asterisk, *F*). Schematics above *in situ* panels indicate heart morphology at each stage, and *hapln1a* expression domain within the heart (blue) V, Ventricle; A, Atrium. Scale bar = 100 μm. (*G–I*) Fluorescent *in situ* hybridization analysis of *hapln1a* (magenta) in *Tg(myl7:lifeActGFP)* transgenic embryos shows *hapln1a* is expressed in myocardial cells at 26hpf. (*J–L*) Fluorescent immunostaining of Hapln1a (magenta) in *Tg(myl7:lifeActGFP)* transgenic embryos demonstrating the protein is secreted into the extracellular space predominantly on the left side of the heart tube (magenta) at 26hpf. (*G* and *J*) Dorsal views. (*H* and *I, K* and *L*) Transverse views. (*M–Q*) Fluorescent immunostaining of Hapln1a (magenta) in *Tg(myl7:lifeActGFP)* transgenic embryos at 50hpf revealing Hapln1a is maintained in the cardiac ECM as looping progresses. (*M–O*) Ventral views. (*P* and *Q*) Transverse views. L, left; R, right, scale bar = 50 μm

To identify candidate genes which modulate cardiac ECM expansion, we took a genome-wide unbiased approach to identify genes expressed in the heart tube at 26hpf, prior to the onset of looping morphogenesis. Since we observed the strongest left-sided ECM expansion in the putative atrium, as well as a generally more expanded ECM at the venous pole of the heart compared to the arterial pole, we used the previously described Tomo-seq technique to generate a regionalized map of gene expression from pole-to-pole in the heart tube^25^^,^^26^ (*Figure 2A*). We sectioned two individual hearts along the atrioventricular axis, identifying 6787 and 8916 expressed genes (Supplementary material online, *Tables S2–S5*), of which approximately half were expressed in more than one section. By identifying which sections express the atrial marker *myh6* (*myosin, heavy chain 6, cardiac muscle, alpha*), we defined a subset of sections with atrial identity. We subsequently filtered genes that were up-regulated in atrial sections compared to ventricular sections in both hearts and examined this list for genes implicated in ECM modification.

Using this approach, we identified *hyaluronan and proteoglycan link protein 1a (hapln1a,* formerly *crtl1)* as a candidate to drive regionalized ECM expansion (*Figure 2B*). The Hapln family of proteins are secreted into the ECM where they cross-link HA to proteoglycans,^37^ suggesting Hapln1a may modify the cardiac ECM environment. mRNA *in situ* hybridization analysis revealed that *hapln1a* is expressed in the posterior of the heart disc and cardiac cone prior to formation of the linear heart tube (*Figure 2C and D*). At 26hpf, *hapln1a* expression is up-regulated on the left side of the heart tube with elevated levels of expression in the future atrium compared to the future ventricle, recapitulating the regionalized ECM expansion in the heart (compare *Figures 2E and* *1K*). This dynamic *hapln1a* expression is in line with recent studies demonstrating that the posterior compartment of the cardiac disc is re-positioned to the left side of the heart tube.^38^ By 50hpf *hapln1a* expression is restricted to low levels in the atrioventricular canal (*Figure 2F*). Fluorescent *in situ* hybridization demonstrates *hapln1a* is expressed in the myocardium (*Figure 2G–I*), while analysis of Hapln1a protein localization confirms it is deposited in the ECM (*Figure 2J–L*). Despite the absence of *hapln1a* expression in the heart at 50hpf (*Figure 2F*), Hapln1a protein is maintained in the ECM at 50hpf (*Figure 2M–Q*), suggesting that the ECM environment established during early stages prior to heart tube formation is maintained during heart development and may be important for subsequent cardiac morphogenesis.

### 3.3 Hapln1a is required for heart morphogenesis and promotes ECM expansion

**Figure 3:**
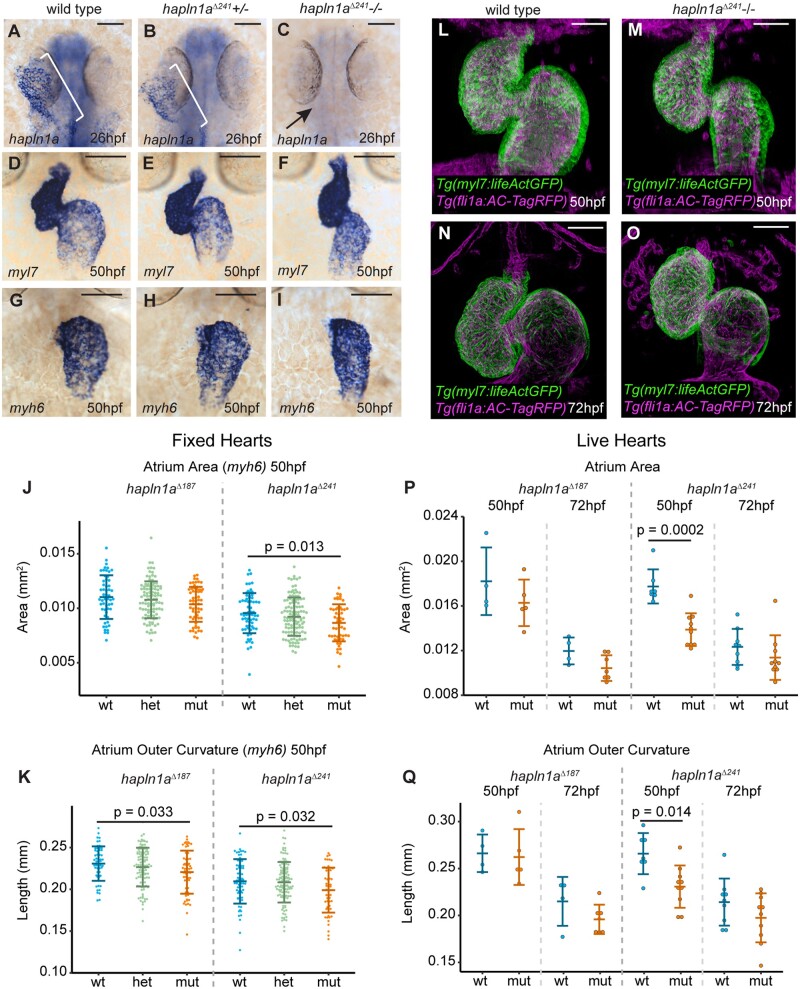
Hapln1a promotes atrial growth. (*A–C*) mRNA *in situ* hybridization analysis of *hapln1a* expression at 26hpf in embryos from an incross of *hapln1a^Δ241^* heterozygous carriers. Wild-type and heterozygous siblings express *hapln1a* in the heart (bracket *A, B*, respectively), whereas *hapln1a* is absent in homozygous mutants (arrow *C*). (*D*–*I*) mRNA *in situ* hybridization expression analysis at 50hpf of *myl7* (*D*–*F*) and *myh6* (*G–I*) in wild-type siblings (*D* and *G*), *hapln1a^Δ241^* heterozygous siblings (*E* and *H*) or *hapln1a^Δ241^* homozygous mutant embryos (*F* and *I*). Scale bar = 50 μm. (*J* and *K*) Quantification of atrial area (*J*), and atrial outer curvature (*K*) in ISH-processed sibling embryos (wt/het) and *hapln1a^Δ241^* or *hapln1a^Δ187^* mutants (mut) at 50hpf. Atrial area is significantly reduced in *hapln1a^Δ241^* mutants compared to wild-type siblings (*P* = 0.013), and atrial outer curvature is significantly reduced in both *hapln1a^Δ187^* and *hapln1a^Δ241^* mutants (*P* = 0.033 and *P* = 0.032). In both *J* and *K, n* = 54 *hapln1a^Δ187^* wt; 104 *hapln1a^Δ187^* het; 60 *hapln1a^Δ187^* mut, 66 *hapln1a^Δ241^* wt; 116 *hapln1a^Δ241^* het; 53 *hapln1a^Δ241^* mut. (*L–O*) Maximum intensity projections of light-sheet z-stacks of live 50hpf (*L* and *M*) and 72hpf (*N* and *O*) *Tg(myl7:lifeActGFP); Tg(fli1a:AC-TagRFP)* transgenic wild-type (*L* and *N*), and *hapln1a^Δ241^* mutant embryos (*M* and *O*). Scale bar = 50μm. (*P* and *Q*) Quantification of atrial area (*P*), and atrial outer curvature (*Q*) in live light-sheet z-projections from wild-type sibling embryos and *hapln1a^Δ241^* or *hapln1a^Δ187^* mutants at 50hpf and 72hpf. Atrial area and atrial outer curvature are significantly reduced in *hapln1a^Δ241^* mutants (mut) compared to wild-type siblings (wt/het) at 50hpf (*P* = 0.0002, and *P* = 0.014). In both (*P*) and (*Q*), *n* = 4 *hapln1a^Δ187^* 50hpf wt; 5 *hapln1a^Δ187^* 50hpf mut, 7 *hapln1a^Δ241^* 50hpf wt; 10 *hapln1a^Δ241^* 50hpf mut; 4 *hapln1a^Δ187^* 72hpf wt; 7 *hapln1a^Δ187^* 72hpf mut, 9 *hapln1a^Δ241^* 72hpf wt; 10 *hapln1a^Δ241^* 72hpf mut. Comparative statistics carried out using a Kruskal–Wallis test with multiple comparisons.

To determine whether Hapln1a is required for cardiac morphogenesis, we used CRISPR-Cas9-mediated genome editing to generate *hapln1a* mutants by deleting the putative *hapln1a* promoter therefore abolishing *hapln1a* expression (Supplementary material online, *Figure S4*). We recovered two alleles; a 187-bp deletion (*hapln1a^Δ187^*) and a 241-bp deletion (*hapln1a^Δ241^*) and established both as stable lines at F2. Both alleles remove the initiating ATG and upstream sequence. To confirm both deletions removed the *hapln1a* promoter and abrogated transcription, *hapln1a* expression was analyzed at 26hpf in F3 mutant embryos. Homozygous *hapln1a* promoter mutants of either allele exhibit a complete loss of *hapln1a* expression at 26hpf compared to wild-type embryos (*Figure 3A–C* and Supplementary material online, *Figure S4*), demonstrating successful deletion of the *hapln1a* promoter. While analysis of heart development in *hapln1a ^Δ241^* mutants at 50hpf by *in situ* hybridization of the pan-cardiac marker *myl7 (myosin light chain 7)* did not reveal striking abnormalities in cardiac morphogenesis (*Figure 3D–F*), we did observe mild heart malformations and an apparent reduction in atrial size. To investigate this further, we quantified heart size and chamber morphology at 50hpf by *in situ* hybridization analysis of *myl7*, the atrial marker *myh6* and the ventricular marker *myh7l* (*myosin heavy chain 7-like*)^39^ (*Figure 3* and Supplementary material online, *Figures S4* and *S5*). We observed a reduction in atrial size in *hapln1a^Δ241^* mutants and a reduction in the length of the outer curvature of the atrium in both *hapln1a^Δ241^* and *hapln1a^Δ187^* mutants, suggesting a defect in atrial growth (*Figure 3J and K*). We performed similar analyses by generating z-projections of live light-sheet images of *Tg(myl7:lifeActGFP); Tg(fli1a:AC-TagRFP)* transgenic wild-type sibling and *hapln1a* mutant hearts at 50hpf and 72hpf, allowing us to assess ongoing heart morphogenesis without chamber morphology being altered during fixation and processing (*Figure 3L–O* and Supplementary material online, *Figure S6*). We confirmed a reduction in atrial size and ballooning of the atrial outer curvature in live *hapln1a^Δ241^* mutants at 50hpf (*Figure 3P and Q*), as well as reductions in atrial perimeter and elongation at 72hpf in *hapln1a^Δ187^* mutants (Supplementary material online, *Figure S6*), with similar trends in reduction in all atrial parameters across alleles. At 72hpf *hapln1a* mutants also display abnormally positioned atria compared to wild-type siblings, with reduced compaction of the heart, and mispositioning of the atrium and ventricle relative to each other (Supplementary material online, *Figure S6*). While expressivity of phenotype appears variable between alleles, both exhibit significant changes in morphological parameters indicative of defective atrial growth. Supporting a requirement in atrial growth specifically after heart tube formation, we did not observe significant defects in heart size or tube position at 26hpf, or in looping morphology, or ventricular growth at 50hpf in *hapln1a* mutants (Supplementary material online, *Figure S5*). Both *hapln1a* mutant alleles are adult viable and we did not observe more profound defects in maternal-zygotic *hapln1a* mutants (data not shown). Together this demonstrates that similar to mouse,^15^  *hapln1a* is required for atrial morphogenesis in zebrafish.

**Figure 4:**
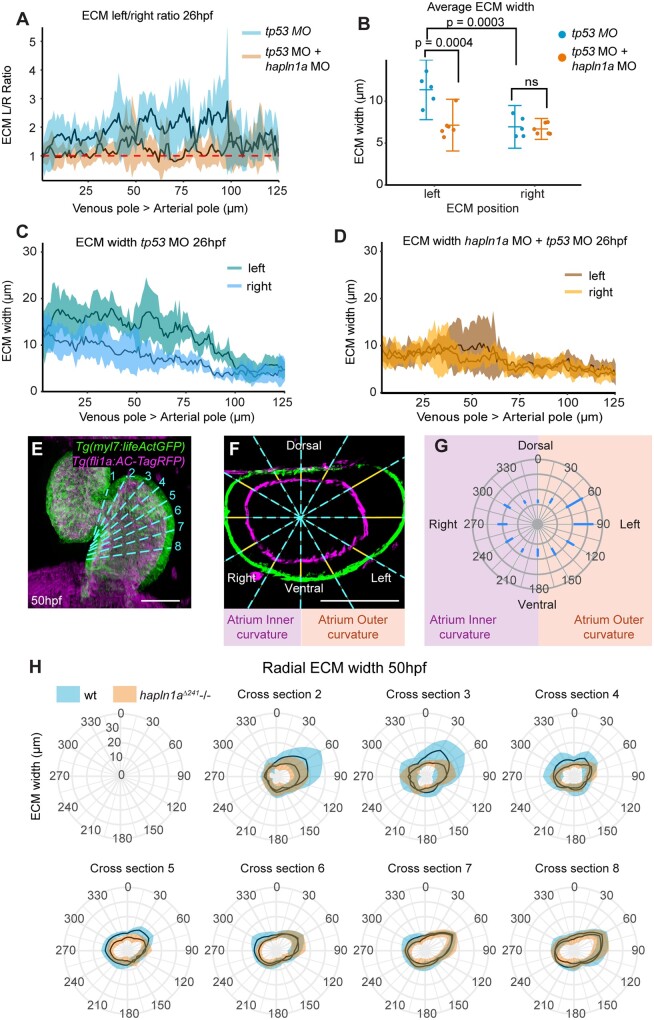
Hapln1a drives regionalized ECM expansion. (*A*) Quantification of ECM left/right width along the longitudinal axis of the heart at 26hpf in embryos injected with either *tp53* MO (blue, *n* = 5) or *hapln1a* MO *+* *tp53* MO (orange, *n* = 6). Mean ± SD are plotted. (*B*) Average ECM width on the left or right side of the heart tube in embryos injected with *tp53* MO (blue, *n* = 5) or *hapln1a* MO *+* *tp53* MO (orange, *n* = 6). *tp53*-injected controls display an expanded ECM on the left side of the heart tube compared to the right, (*P* = 0.0003, 2-way ANOVA) whereas embryos injected with *hapln1a* MO *+* *tp53* MO have a reduced left ECM size when compared to *tp53* MO injected (*P* = 0.0004), resulting in loss of left-sided ECM expansion. Mean ± SD are plotted. (*C* and *D*) Quantification of ECM width on the left and right sides of the heart tube from venous pole to arterial pole at 26hpf in embryos injected with *tp53* MO (*C, n* = 5) or *hapln1a* MO *+* *tp53* MO (*D, n* = 6). Mean ± SD are plotted. The cardiac ECM in *tp53* morphants exhibits atrial and left side expansion, whereas the ECM in *hapln1a* morphants is more uniform in width from atrium to ventricle and is not expanded on the left side. Mean ± SD are plotted. (*E*) Maximum intensity projection of light-sheet z-stacks of 50hpf *Tg(myl7:lifeActGFP); Tg(fli1a:AC-TagRFP)* transgenic embryo. Dashed cyan line indicates position of optical cross-sections. (*F*) Example orthogonal view through the atrium of wild-type embryo in (*E*). Blue dashed lines indicate radial positions for measuring ECM thickness (yellow lines). Scale bar = 50μm. (*G*) Schematic of radial plot corresponding to radial ECM positions in (*F*). (*H*) Quantification of ECM width in atrial cross-sections at defined angular positions along the longitudinal axis of the atrium from AVC (cross-section 2) towards the venous pole (cross-section 8) at 50hpf in wild-type (blue) or *hapln1a^Δ241^* mutants (orange). Asymmetric ECM width is reduced in the outer curvature close to the AVC (cross-sections 2–4) in *hapln1a^Δ241^* mutants compared to wild-type siblings. Mean ± SD are plotted, radial-axis is consistent between plots, *n* ≥ 4 at each location.

Hapln1 functions as an ECM binding protein and its localization recapitulates the regionalized ECM expansion in the heart tube, therefore, we hypothesized that Hapln1a promotes cardiac morphogenesis by driving regionalized ECM expansion in the heart. Since both *hapln1a* promoter deletion alleles carry the *Tg(myl7:lifeActGFP)* transgene, this prevented analysis of ECM width throughout the heart tube of *hapln1a* mutants using the *ssNcan-GFP* HA sensor. We instead injected a morpholino (MO) against *hapln1a* into zebrafish embryos at the 1-cell stage together with a *tp53* MO control and the *ssNcan-GFP* HA sensor and assessed ECM expansion in the heart tube at 26hpf. Analysis of Hapln1a protein levels in *hapln1a* morphants confirms successful blocking of Hapln1a translation in the morphants (Supplementary material online, *Figure S7*). Control embryos injected with *tp53* MO demonstrate the regionalized ECM expansion previously observed, with a left-sided expansion of the ECM (*Figure 4A and B*), and a higher level of ECM expansion in the atrium vs. the ventricle (*Figure 4C*), although overall ECM width was slightly reduced compared to uninjected embryos (compare *Figures 4C and 1N*). Embryos injected with *hapln1a* MO *+* *tp53* MO did not exhibit either atrial or left-sided ECM expansion (*Figure 4A, B, and D*), suggesting that Hapln1a drives regionalized ECM expansion in the heart tube. To confirm a role for Hapln1a in ECM regionalization, we analyzed ECM size in a series of optical cross-sections from live light-sheet images of wild-type and *hapln1a* mutant *Tg(myl7:lifeActGFP); Tg(fli1a:AC-TagRFP)* transgenic embryos, taking sections from the atrioventricular canal through the atrium towards the venous pole at 50hpf and 72hpf (*Figure 4E–H* and Supplementary material online, *Figure S8*). Analysis of radial ECM width in the atrium of *hapln1a* mutants revealed a reduction in ECM specifically in the outer curvature of the atrium proximal to the AVC (*Figure 4H* and Supplementary material online, *Figure S8*, cross-sections 2–4), where ECM expansion is most profound in wild-type hearts and defects in atrial morphology occur in *hapln1a* mutants (*Figure 3K and Q*). Together this supports a role for Hapln1a in regionally regulating cardiac ECM size to promote normal cardiac morphogenesis.

### 3.4 Hapln1a and HA interact to drive heart morphogenesis

Hapln1a is a member of a family of ECM binding proteins which cross-link HA with proteoglycans.^37^ Since *hapln1a* is transiently expressed during early heart development at cardiac disc and early tube stage (*Figure 2*), this suggests that the cardiac ECM driving continued morphogenesis of the heart is established at early stages of heart development and requires the interaction of Hapln1a with HA. To interrogate the temporal requirements for HA in heart looping, we applied the HA synthesis inhibitor 4-methylumbelliferone (4-MU^40^^,^^41^) to embryos prior to the onset of heart tube formation at 18hpf, and either washed the drug off at 22hpf, or left the embryos to develop to 48hpf, when we assessed heart morphology. Inhibiting HA synthesis from cardiac disc stage (18hpf) until 48hpf often arrested heart development mid-way during tube formation (Supplementary material online, *Figure S9*), a more profound phenotype than that observed in *has2* zebrafish morphants or *Has2* mouse mutants.^17^^,^^18^ However, inhibition of HA synthesis during the short time window between cardiac disc (18hpf) and cardiac cone (22hpf) stage, prior to tube formation, resulted in normal tube formation but a specific disruption to subsequent cardiac looping morphogenesis (Supplementary material online, *Figure S9*). This supports the hypothesis that HA synthesized prior to formation of the heart tube is required for ongoing morphogenesis of the heart.

Having demonstrated a requirement for HA synthesis in heart morphogenesis during early cardiac development when *hapln1a* expression is initiated, we wanted to confirm the interaction of *hapln1a* and HA in heart looping morphogenesis. Injection of sub-phenotypic doses of morpholinos targeting either *has2* or *hapln1a* did not result in significant defects in cardiac morphology at 48hpf (Supplementary material online, *Figure S9*). However, co-injection of both *has2* and *hapln1a* morpholinos results in profound defects in heart development at 48hpf (Supplementary material online, *Figure S9*), including a reduction in heart looping ratio, and abnormal atrial morphology. This is a more severe phenotype than that observed by either injection of *hapln1a* MO *+* *tp53* MO*, has2* MO *+* *tp53* MO, 4MU treatment or deletion of the *hapln1a* promoter, suggesting that while timely HA synthesis drives heart morphogenesis subsequent to tube formation, *hapln1a* is an important regional modulator of this process.

### 3.5 Hapln1a misexpression results in abnormal heart morphogenesis

**Figure 5:**
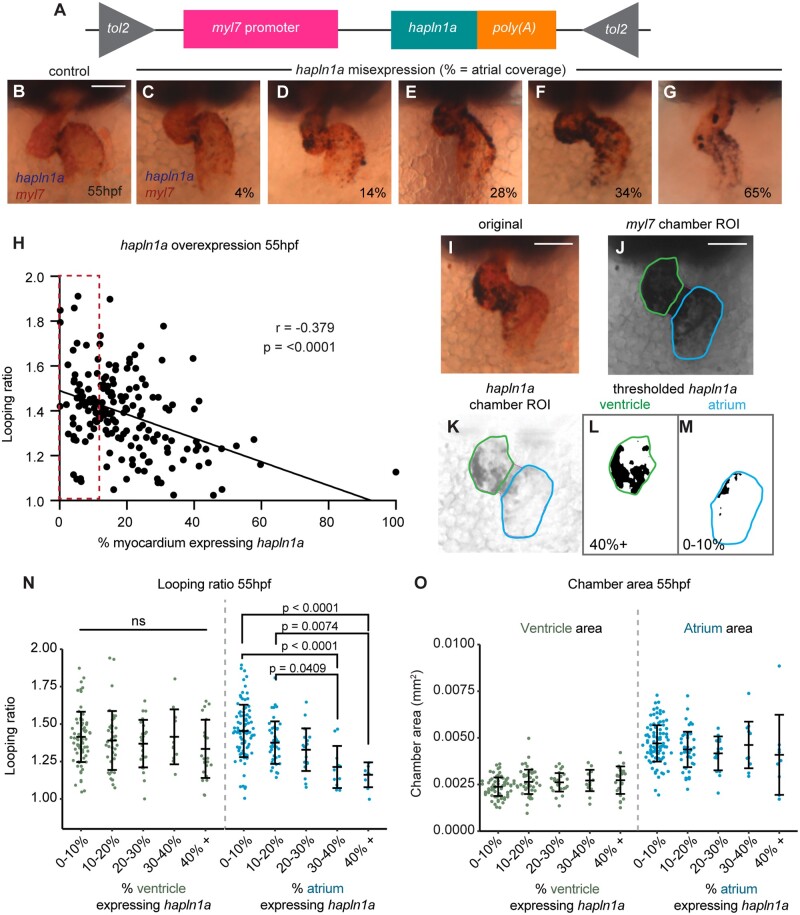
Regionalized *hapln1a* expression in the atrium promotes heart morphogenesis. (*A*) Schematic of DNA construct used to misexpress *hapln1a* specifically in cardiomyocytes. (*B–G*) Example images of heart morphology (*myl7*, red) upon different levels of *hapln1a* misexpression (blue) at 55hpf, related to the quantification method depicted in (*I–M*). Percentage indicates *hapln1a* coverage of the atrium. (*H*) Scatter plot depicting looping ratio as a function of percentage of the heart covered by *hapln1a* expression together with linear regression of the data (*n* = 174). Spearman’s correlation coefficient (*r*) deviates significantly from zero (*P* < 0.0001) demonstrating that increased coverage of *hapln1a* in the myocardium results in reduced heart looping morphogenesis. Red dashed box indicates embryos with wild-type levels of *hapln1a* expression. (*I*–*M*) Quantification approach to analyze pan-cardiac or chamber-specific *hapln1a* misexpression at 55hpf. The number of *hapln1a*-positive pixels within each chamber is measured alongside the total chamber area, quantifying the percentage of the chamber expressing *hapln1a*. (*N–O*) Analysis of looping ratio (*N*) and chamber area (*O*) as a function of the level of *hapln1a* expression in each chamber of the heart. Embryos are categorized depending on the percentage of the chamber expressing *hapln1a*. Misexpression of *hapln1a* in the ventricle does not affect looping ratio, whereas misexpression of *hapln1a* in the atrium significantly reduces looping ratio at ≥30% coverage (*N*). *hapln1a* misexpression in either chamber does not appear to alter overall heart size (*O*). ns = not significant. In both (*N*) and (*O*), atrium misexpression categories: *n* = 92 (0–10%); 43 (10–20%); 17 (20–30%); 12 (30–40%); 9 (40+%); ventricle misexpression categories: *n* = 73 (0–10%); 41 (10–20%); 25 (20–30%); 12 (30–40%); 23 (40+%). Comparative statistics carried out using a Kruskal–Wallis test with multiple comparisons.

While analysis of *hapln1a* mutants demonstrates a requirement for Hapln1a in heart development (*Figure 3* and Supplementary material online, *Figure S6*), we wished to investigate whether the regionalization of *hapln1a* expression is important for cardiac morphogenesis. We generated a DNA construct in which the full-length *hapln1a* coding sequence is driven by the pan-myocardial *myl7* promoter, flanked by Tol2 transposon sites to allow integration into the genome (*Figure 5A*). We co-injected *myl7*: *hapln1a* DNA with *tol2* transposase mRNA at the 1-cell stage and analyzed both *myl7* and *hapln1a* expression at 55hpf, allowing us to visualize heart morphology alongside assessing the extent of *hapln1a* misexpression (*Figure 5B–G*). We analyzed heart morphology by quantifying looping ratio and assessed this as a function of percentage coverage of *hapln1a* expression in the whole heart (*Figure 5H*). Increasing the domain of *hapln1a* expression in the heart results in a reduction in looping morphogenesis (*Figure 5H*), suggesting that regionalized expression of *hapln1a* in the heart is important for cardiac morphogenesis. Since *hapln1a* expression and ECM asymmetry is greater in the atrium than the ventricle, we hypothesized that *hapln1a* misexpression in each chamber may impact differently on heart morphogenesis. We quantified *hapln1a* misexpression in each chamber by calculating the percentage of the chamber which expresses *hapln1a* (*Figure 5I–M*), and found that while misexpression of *hapln1a* in the ventricle did not impact upon heart morphogenesis, misexpression of *hapln1a* in the atrium decreased looping ratio (*Figure 5N*). Conversely, we did not find significant changes in chamber area upon *hapln1a* overexpression (*Figure 5O*). Together with our observations of abnormal heart morphology in *hapln1a* mutants this suggests that spatially restricted *hapln1a* expression in the atrium drives cardiac morphogenesis.

### 3.6 Embryonic left–right asymmetry orients the axis of ECM asymmetry in the heart tube

**Figure 6:**
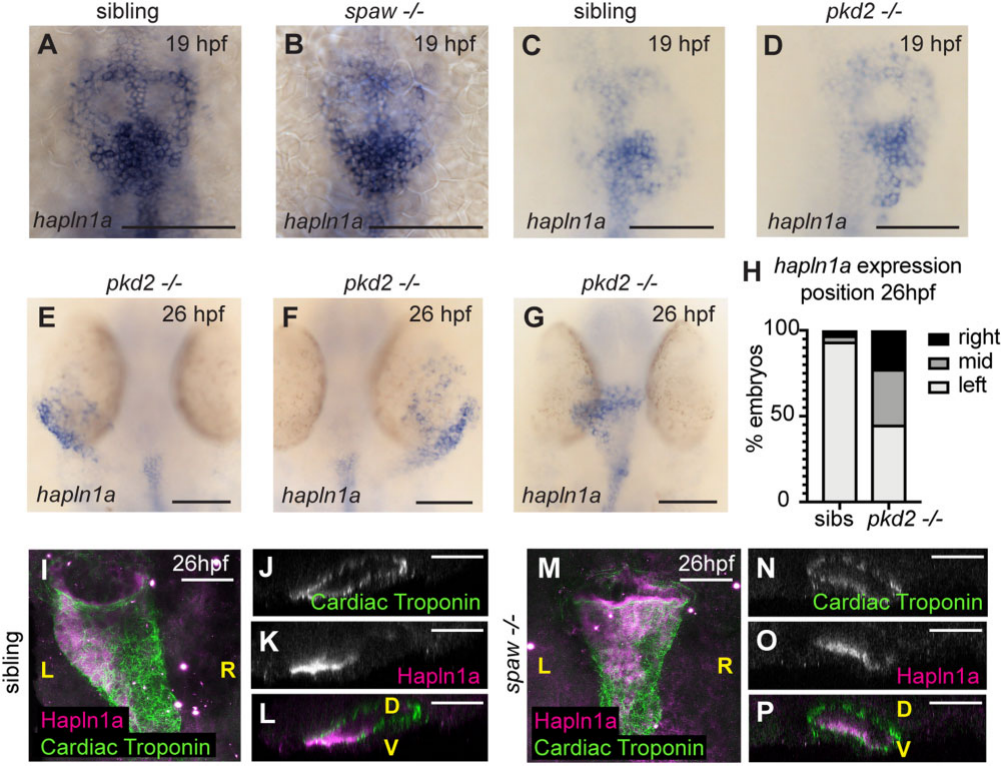
Posterior up-regulation of *hapln1a* in the cardiac disc is independent of left–right asymmetry. (*A–G*) mRNA *in situ* hybridization analysis of *hapln1a* expression in an incross of *spaw* (*A* and *B*) or *pkd2^hu2173^* (*C–G*) heterozygous carriers. At 19hpf *hapln1a* is expressed in the posterior cardiac disc of both *spaw* mutant embryos (*B, n* = 17/19), and *pkd2* mutant embryos (*D, n* = 13/13), similar to sibling embryos. At 26hpf *pkd2* mutant hearts that have jogged to the left exhibit left side up-regulation of *hapln1a* (*E, n* = 18), *pkd2* mutant hearts on the right have right side up-regulation of *hapln1a* (*F, n* = 9), and *pkd2* mutant hearts that remain at the midline have no clear left–right asymmetry in expression (*G, n* = 13). (*H*) Quantification of position of *hapln1a* expression in sibling and *pkd2^hu2173^* mutant embryos at 26hpf, *n* = 138 *pkd2^hu2173^* siblings, 40 *pkd2^hu2173^* mutants. (*I–P*) Fluorescent immunostaining of Hapln1a (magenta) and cardiac troponin (green) at 26hpf in wild-type siblings (*I–L*) or *spaw* mutant embryos (*M–P*). Wild-type siblings exhibit left-sided deposition of Hapln1a in the heart tube (*I–L, n* = 6), whereas *spaw* mutant embryos exhibit ventral localization of Hapln1a (*M–P, n* = 6). (*I* and *M*) Dorsal views. (*J–L* and *N–P*) Optical transverse sections. Scale bar = 50μm. L, left; R, right; D, dorsal; V, ventral.

Finally, since *hapln1a* is asymmetrically expressed on the left side of the heart tube, and is required for heart morphogenesis, we hypothesized that it may contribute to a previously-described tissue-intrinsic mechanism of heart looping morphogenesis^7^ and thus is expressed independent of embryonic left–right asymmetry cues. Embryos with mutations in *pkd2 (polycystic kidney disease 2*), which is required for Kupffer’s Vesicle function,^42^ exhibit defects in left–right asymmetry including a disruption to normal leftward displacement of the heart tube,^42^ while *spaw* mutants lack asymmetric Nodal expression prior to asymmetric organ morphogenesis resulting in midline positioning of the heart tube.^7^ We hypothesized that induction of *hapln1a* expression occurs independent of embryonic laterality cues, but that asymmetric positioning of *hapln1a*-expressing cells in the heart tube may be tightly linked to the direction of heart tube position, and therefore dictated by embryonic left–right asymmetry. We analyzed *hapln1a* expression in an incross of *pkd2^hu2173^* and *spaw* heterozygotes and found that consistent with our hypothesis *hapln1a* is always expressed in the posterior of the heart disc in both *pkd2^hu2173^* mutants and *spaw* mutants at 19hpf (*Figure 6A–D*). Importantly, at 26hpf, we observed that positioning of *hapln1a*-expressing cells is dependent upon cardiac position—in *pkd2^hu2173^* mutants where the heart is positioned to the right, *hapln1a* is up-regulated on the right side of the tube, whereas if the heart remains midline, *hapln1a* does not exhibit a clear left–right asymmetry in up-regulation (*Figure 6E–H*). Similarly, analysis of Hapln1a deposition in *spaw* mutants at 26hpf reveals Hapln1a is no longer positioned on the left side of the heart tube, but instead is secreted into the cardiac ECM on the ventral face of the heart (*Figure 6I–P*). These data support a model where laterality cues do not initiate *hapln1a* expression but are required for its subsequent position in the heart tube.

Together, this supports a model where initiation of *hapln1a* expression in the posterior cardiac disc is independent of laterality cues, but the subsequent cell movements which occur during heart tube formation reposition this population of cells to the left side of the heart, dictating the axis of ECM asymmetry in the heart tube.

## 4. Discussion

**Figure 7:**
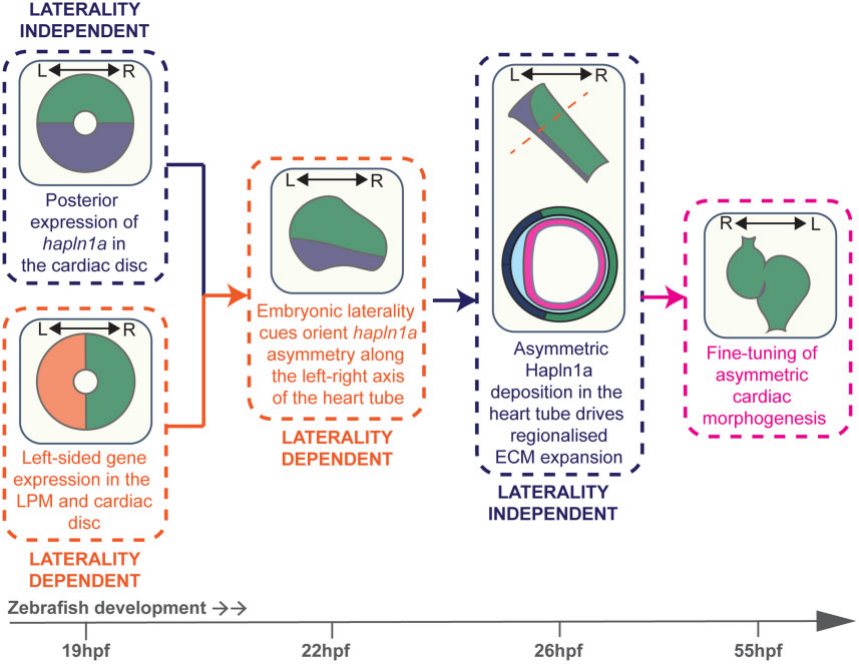
Left–right asymmetry orients ECM asymmetry in the heart tube to fine tune cardiac morphogenesis. Model depicting the interaction between embryonic laterality and ECM regionalization in cardiac morphogenesis. LPM, lateral plate mesoderm; L, left; R, right.

The process of forming the complex heart from a simple linear tube requires careful coordination of spatially restricted extrinsic signals and highly regionalized changes in cell shape and tissue growth to ensure correct shaping of the developing tissue. Our data show that the zebrafish heart tube exhibits regionalized ECM expansion that is dependent upon localized expression of the ECM binding protein *hapln1a* and this promotes cardiac morphogenesis. Our finding that while the onset of *hapln1a* in the posterior cardiac disc is independent of embryonic left–right asymmetry, laterality cues are required to orient asymmetric Hapln1a deposition along the left–right axis of the heart tube, allows us to propose a new model in which heart-extrinsic embryonic asymmetry orients tissue-intrinsic cardiac ECM asymmetry, ensuring that directionality and growth of the heart are tightly coordinated to fine tune cardiac morphogenesis (*Figure 7*).

Interestingly, the only other gene thus far identified specifically in the posterior cardiac disc/left heart tube in zebrafish is *meis2b.*^38^  *meis2b* mutants exhibit defects in atrial morphology at juvenile and adult stages, supporting our conclusion that early anterior–posterior asymmetry in the heart disc/left–right asymmetry in the heart tube are important for continual cardiac morphogenesis. However, contrary to our study which reveals a reduced atrial size in *hapln1a* mutants, *meis2b* mutant adult zebrafish exhibit an enlarged atrium,^38^ suggesting these two genes play opposing roles in atrial morphogenesis. Investigating *hapln1a* expression in *meis2b* mutants may uncover any interactions between these two genes in heart development. Similarly, *hapln1a* mutants are adult viable, and comparing atrial phenotypes in juveniles and adults will better define the phenotypic relationship between these two genes, as well as the impact of abnormal embryonic morphology on adult heart form and function. Furthermore, recent studies have shown that cross-talk between the myocardium and endocardium modulates atrial growth,^43^ and our study suggests that differential ECM composition and/or degradation may help regionally fine tune this process to dictate chamber morphology.

The mechanism by which regionalized ECM composition modulates cardiac morphogenesis remains unclear. A major role of the ECM in tissue morphogenesis is to provide structural or biomechanical cues to neighbouring tissues. Alternatively, Hapln1a-mediated cross-linking may modulate regional stiffness of the cardiac ECM, and differential matrix stiffness has been shown to regulate cardiomyocyte form and function.^44–47^ Importantly, we do not see defects in heart rate in *hapln1a* mutants (Supplementary material online, *Figure S5*), suggesting this regionalized ECM is not regulating cardiac function. In addition to provision of mechanical cues to the surrounding cells, the ECM also modulates diffusion and availability of extracellular signalling molecules.^48^ It is therefore tempting to speculate that the specific ECM environment allows precise regionalized cellular responses to pan-cardiac or chamber specific signalling pathways.

Hapln proteins cross-link HA to proteoglycans. HA and Versican have previously been implicated in heart development, however global loss of these genes throughout the heart result in profound morphological defects.^17^^,^^19^ We suggest that regionalized modification of the ECM by Hapln1a allows the developing heart to generate different regional responses to globally deposited ECM components, resulting in spatial fine-tuning of the specific morphological rearrangements required for complex tissue shaping. While regional ECM cross-linking may change the biomechanical properties of the ECM by stabilizing these components in specific regions of the heart tube, HA and proteoglycan cleavage products can also act as signalling molecules.^49^ Our finding that *hapln1a* and *has2* work synergistically to promote heart morphogenesis suggests that Hapln1a could promote regional atrial growth by regulating HA signalling in the heart.

Importantly, while our data show that ECM regionalization appears to be the result of asymmetric *hapln1a* expression and deposition, other factors may support and maintain ECM regionalization during development. Although we do not observe asymmetric *has2* expression, we cannot rule out asymmetries in deposition which current analytical methods are not sensitive enough to detect. Similarly, we cannot discard the possibility that asymmetric activity of additional ECM modifiers or hyaluronidases enhance ECM regionalization through localized degradation, and there is some evidence that hyaluronidases themselves are regionally localized in the heart tube.^50^ This suggests multiple regulatory mechanisms may interact to regionalize HA/ECM activity in the developing heart. Comparative quantitative analysis of cardiac morphology in single and combinatorial knockout models will help define how ECM components and modifiers interact to contribute to heart morphogenesis. Our relatively simplistic analysis of 3D chamber morphology suggests that 2D analyses do not capture all aspects of cardiac morphology during development, and thus defining the links between spatiotemporal ECM dynamics and cardiac morphology requires better definition of changes in ECM composition and morphological cardiac parameters in 3D.

Additionally, *Hapln1* mouse mutants exhibit decreased protein levels of the proteoglycan Versican,^15^ suggesting that Hapln1-mediated HA-Versican cross-linking prevents degradation of one or both of these components. Further supporting an interaction between Hapln1a, Versican, and HA in heart morphogenesis, both mice and medaka lacking Versican exhibit severe cardiac malformations.^19^^,^^36^ Versican proteins can be subject to cleavage by ADAMTS proteases,^51^ depending on isoform and domain structure, and reduction in ADAMTS protease activity results in reduced Versican cleavage and cardiac abnormalities.^52^^,^^53^ We have shown that of the two zebrafish versican paralogs, only *vcana* expression overlaps the *hapln1a* expression domain (Supplementary material online, *Figure S3*). Zebrafish Vcana appears to be a small V3 or V4-like isoform which is not predicted to undergo cleavage (Uniprot ID A0A2R8Q3K1). This suggests that in zebrafish Hapln1a may not stabilize Versican in the ECM, and alternatively Hapln1a cross-linking may promote regional degradation of HA in the heart tube.

*Hapln1* mutant mouse embryos also exhibit relatively mild structural cardiac malformations consistent with abnormal early cardiac morphogenesis.^15^ However, while that study describes *Hapln1* expression in the valve leaflets it does not address a potentially conserved role for transiently asymmetric *Hapln1* expression during earlier heart development. Zebrafish have two *hapln1* paralogs, each with a distinct expression profile in the heart during development, with *hapln1b* expressed primarily in the endocardium (data not shown). Thus, zebrafish provide an opportunity to define tissue-specific requirements for Hapln1 function in either the myocardium or endocardium during cardiac morphogenesis.

Together this study identifies a novel functional role for ECM regionalization in the developing heart, mediated by the HA cross-linking protein Hapln1a, and provides a new model in which ECM regionalization acts together with embryonic laterality cues to drive cardiac morphogenesis.

## Supplementary material

Supplementary material is available at *Cardiovascular Research* online.

## Authors’ contributions

C.J.D. and E.S.N. conceived the study and designed the experiments. C.J.D., J.S.-P., F.H., E.J.G.P., and E.S.N. carried out experimental work. A.M.S., R.N.W., and T.J.C. shared the *Tg(fli1a: AcTagRFP)* transgenic line prior to publication. F.T. and J.B. generated the *Tg(lft2BAC: Gal4FF)* transgenic line, and F.J.v.E. characterized the *pkdhu2173* allele. J.B. provided financial support for the Tomo-seq experiments. E.S.N. wrote the manuscript with input from C.J.D., J.S.-P., E.J.G.P., F.T., and T.J.C.

## Supplementary Material

cvab004_Supplementary_Data

## Notes

Translational Perspective This study reveals that the cardiac ECM exhibits regional specialization required for heart morphogenesis, and sheds light on how embryonic left–right asymmetry acts in concert with ECM regionalization to fine tune heart shape. This work can help us understand the origins of congenital heart defects, and in particular the nature of morphological heart abnormalities in patients with heterotaxia-associated heart malformations. Furthermore, recent studies suggest the ECM is a key regulator of regenerative potential in the heart, thus defining how distinct ECM composition impacts upon heart form and function has implications for developing regenerative therapies in the future.
