# Supplementary material for: Asymmetric Hapln1a drives regionalized cardiac ECM expansion and promotes heart morphogenesis in zebrafish development
Source: Cardiovasc Res. 2021 Feb 22;118(1):226–40. doi: 10.1093/cvr/cvab004 (PMC8752364; doi:10.1093/cvr/cvab004)
Supplement: cvab004_Supplementary_Data [file cvab004_supplementary_data.zip › Derrick Sanchez Posada Supplemental Data Resubmission 3.pdf]

CVR-2019-1247R1

Supplementary Material

Asymmetric Hapln1a drives regionalised cardiac ECM expansion and promotes heart morphogenesis in zebrafish development

Christopher J Derrick[*], Juliana Sánchez-Posada[*], Farah Hussein, Federico Tessadori, Eric JG Pollitt, Aaron M Savage, Robert N Wilkinson, Timothy J Chico, Fredericus J van Eeden, Jeroen Bakkers, Emily S Noël[+].

[*] These authors contributed equally to this work

[+] Corresponding author: Email: e.s.noel@sheffield.ac.uk

Supplementary Figures

Figure S1: **Generation of the *Tg(lft2BAC:Gal4FF)* transgenic zebrafish**

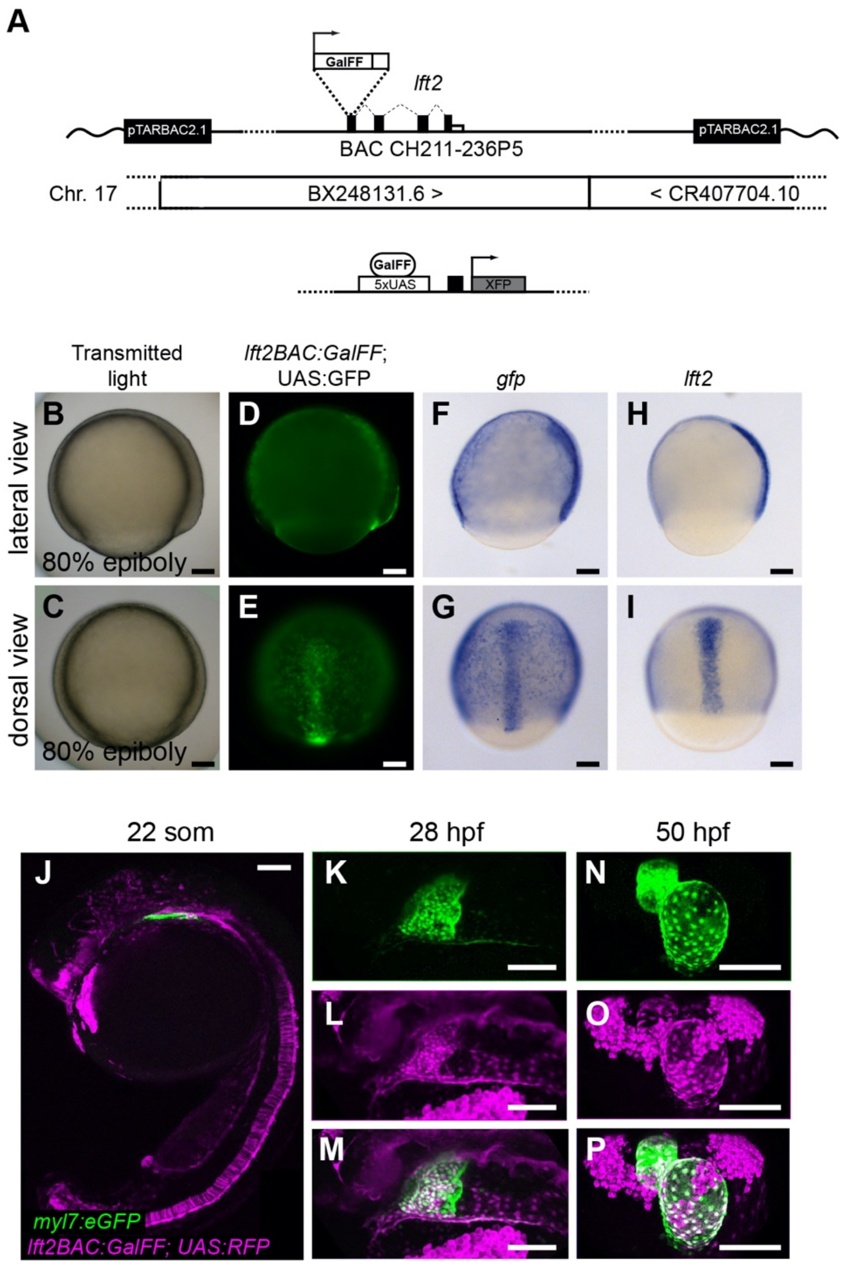

Characterisation of the *Tg(lft2BAC:Gal4FF)* reporter line. (A) An expression cassette containing Gal4FF was recombined into BAC CH211-236P5 at the ATG site of the 1st exon of the *lft2* gene. B-E: Brightfield images (B, C) and corresponding GFP expression domain (D, E) of the *Tg(lft2BAC:Gal4FF)*; *Tg(UAS:GFP)* double transgenic embryos at 80% epiboly. F-G: mRNA *in situ* hybridisation analysis of *gfp* mRNA (F,G). (H-I) mRNA *in situ* hybridisation analysis of *lft2* expression at 80% epiboly. Note the overlap of expression at the forming

midline (compare E, G, I). (J-P) Reporter expression (RFP) in *Tg(lft2BAC:Gal4FF); Tg(UAS:RFP)*; *Tg(myl7:eGFP)* triple transgenic embryos at 22 somites (lateral view, J) and in the cardiac region at 28 hpf (lateral view, K-M) and at 50 hpf (frontal view, N-P). Scale bars = 100μm

Figure S2: Cardiac jelly in the atrium is asymmetrically expanded at 50hpf

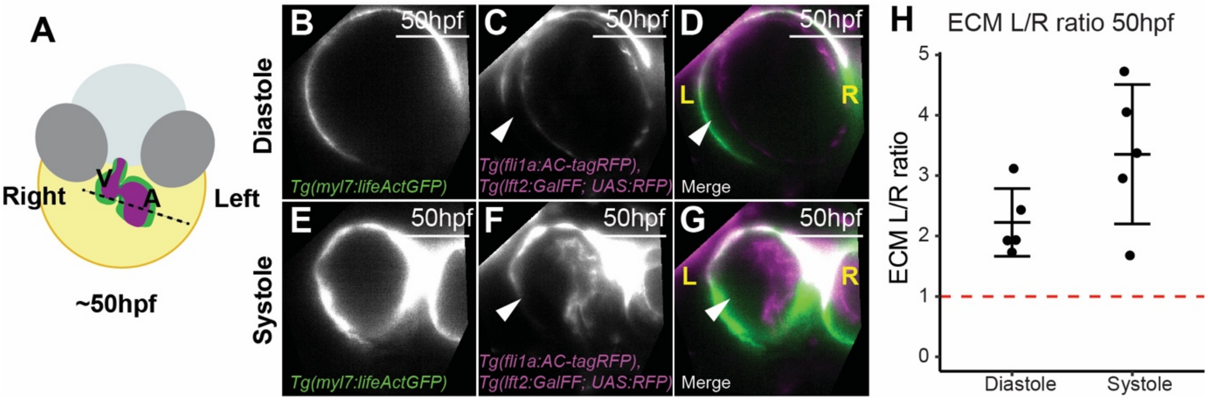

A: Schematic representing the positioning of the heart in a zebrafish embryo at 50hpf (ventral view). Dotted line demarcates the optical transverse sections imaged in panels B-G. B-G: Light-sheet optical cross-sections through the atrium of a 50hpf *Tg(myl7:lifeActGFP); Tg(fli1a:AC-TagRFP); Tg(lft2BAC:Gal4FF); Tg(UAS:RFP*) transgenic embryo during diastole (B-D) and systole (E-G). The myocardium is highlighted in green (B, D, E, G), and the dorsal myocardium and endocardium are highlighted in magenta (C, D, F, G). The extracellular space between the myocardium and endocardium is expanded on the left side of the atrium (white arrowhead). Scale bar = 50µm. H: Quantification of left-right ECM ratio in the atrium at 50hpf, where a value greater than 1 (red dotted line) denotes a left-sided expansion (n=6).

Figure S3: The ECM constituents HA, *versican* and *aggrecan,* and the ECM synthases *has2* and *chsy1* are not asymmetrically expressed in the heart tube

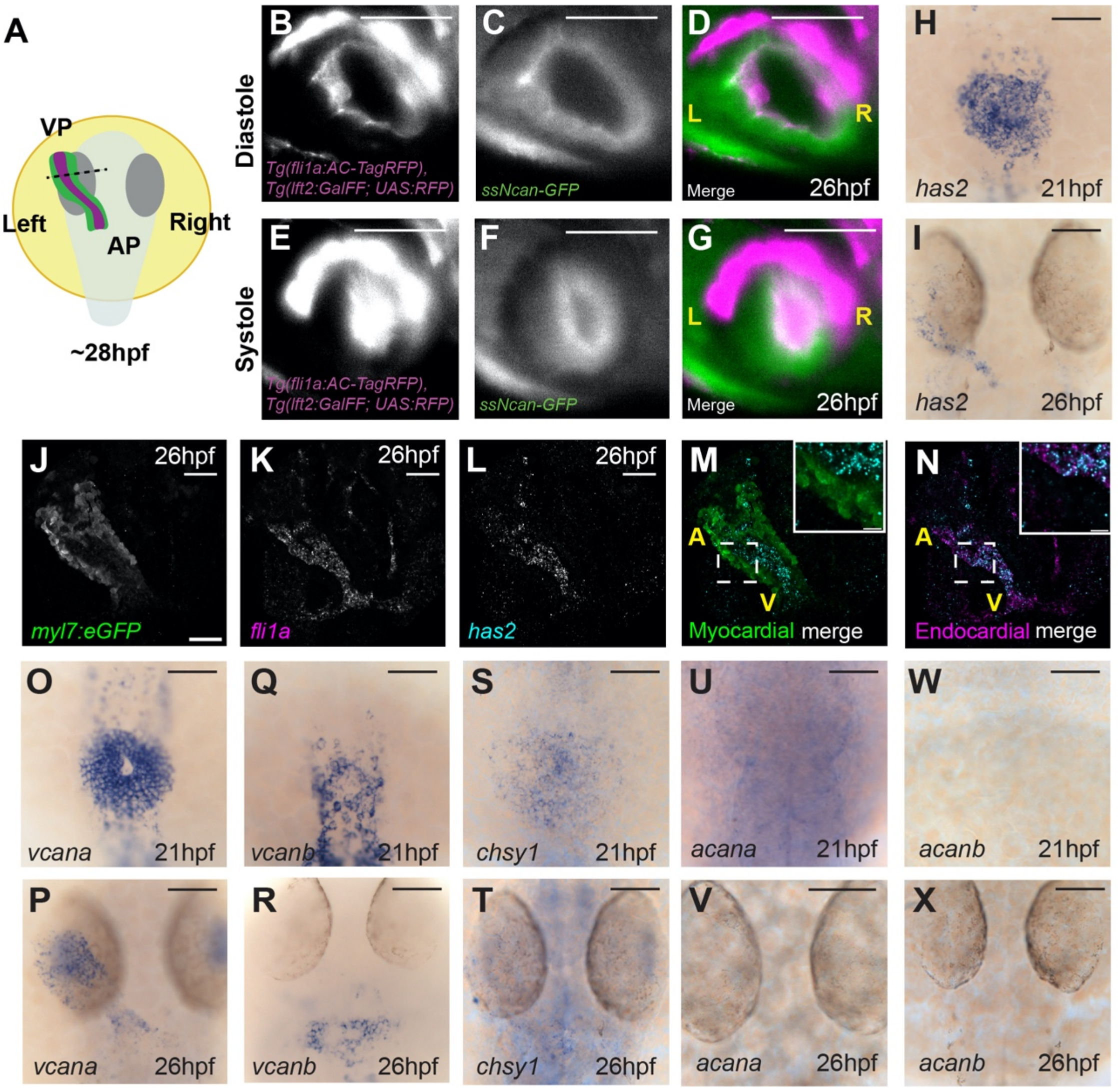

A: Schematic representing the positioning of the heart in a zebrafish embryos at 28hpf (dorsal view), with the dotted line representing the optical transverse sections imaged in panels B-G. B-G: Light-sheet optical cross-sections through the heart tube of a 28hpf *Tg(fli1a:AC-TagRFP); Tg(lft2BAC:Gal4FF); Tg(UAS:RFP)* transgenic embryo injected with *ssNcan-GFP* RNA, imaged during diastole (B-D) and systole (E-G). The *ssNcan-GFP* sensor is highlighted in green (C, D, F, G), and the dorsal myocardium and endocardium are highlighted in magenta (B, D, E, G). The *ssNcan-GFP* binds to HA and is found in the extracellular space between the myocardium and endocardium and does not appear to display asymmetric distribution. L – left,

R – right. Scale bar = 50µm H-I: mRNA *in situ* hybridisation analysis of *has2* reveals it does not display anterior-posterior asymmetry in the heart disc at 21hpf (H) or left-right asymmetry in the tube at 26hpf (I). J-N: Single slice images from two-colour fluorescent mRNA in situ hybridisation analysis of *has2* (L) and *fli1a* (K) expression in *Tg(myl7:GFP)* transgenic embryos (J) at 26hpf confirms that *has2* colocalises with *fli1a* in the endocardium (N). O-X: mRNA *in situ* hybridisation analysis of *vcana* (O, P), *vcanb* (Q, R), *chsy1* (S, T), *acana* (U, V), and *acanb* (W, X) at 21hpf and 26hpf respectively. *vcana* is broadly expressed in the cardiomyocytes of the heart disc at 21hpf, (O) and in the heart tube at 26hpf. (P) Conversely at 21hpf *vcanb* is expressed only in a small ring of cells at the centre of the heart disc (Q), which at 26hpf constitute the outflow tract of the heart tube (R). *chsy1* is expressed at low levels in the heart disc at 21hpf (S), but appears to be absent in the heart tube at 26hpf (T). Neither *aggrecan* paralogs are detected in the heart at these stages (U-X). None of the genes analysed display anterior-posterior asymmetry in the heart disc or left-right asymmetry in the heart tube. H-X: Dorsal views, anterior to top. Scale bar = 50µm

Figure S4: Generation of *hapln1a* mutants and quantification of heart morphology

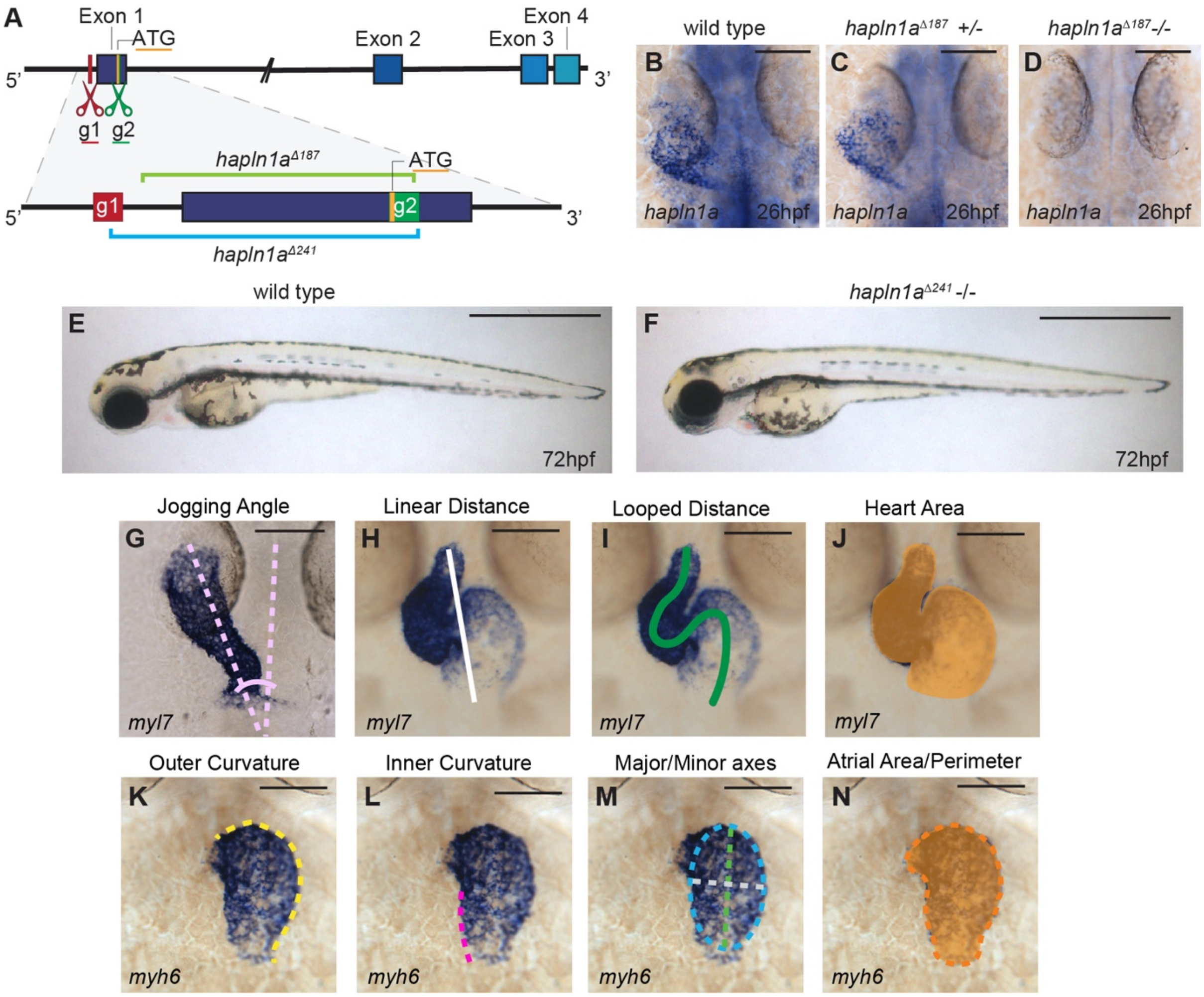

A: Schematic of deletion of putative promoter in *hapln1a* mutants by CRISPR-Cas9 mediated genome editing, based on Ensembl GRCz11 *hapln1a* transcript ID ENSDART00000122966.4. B-D: mRNA *in situ* hybridisation analysis of *hapln1a* expression at 26hpf in embryos from an incross of *hapln1a*$^{\Delta 187}$ heterozygous carriers. Wild type and heterozygous siblings express *hapln1a,* whereas *hapln1a* is absent in homozygous mutants (D). Scale bar = 50µm. E-F: Brightfield images of wild type siblings (E) and *hapln1a*$^{\Delta 241}$ mutants (F) at 72hpf. *hapln1a*$^{\Delta 241}$ mutants do not exhibit any gross morphological defects. Scale bar = 500µm. G-J: Illustration of methods to quantify jogging angle (G, pink angle), linear heart distance (H, length of white line), looped heart distance (I, length of green line) and heart area (J, orange shaded area) from *myl7* mRNA *in situ* hybridisation expression analysis at 26hpf (G) and 50hpf (H-I). Looping

ratio is the quotient of the looped and linear distances. K-N: Illustration of method to quantify atrial outer curvature (K, length of yellow dashed line), atrial inner curvature (L, length of magenta dashed line), atrial major and minor axes (M, fitted elipse - blue dashed line, major axis - length of green dashed line, minor axis - length of white dashed line) and atrial area and perimeter (N, orange shaded area and length of orange dashed line, respectively) from *myh6* mRNA *in situ* hybridisation expression analysis at 50hpf. Scale bar = 50μm.

Figure S5 - *hapln1a* is dispensable for heart jogging, ventricle size and heart function.

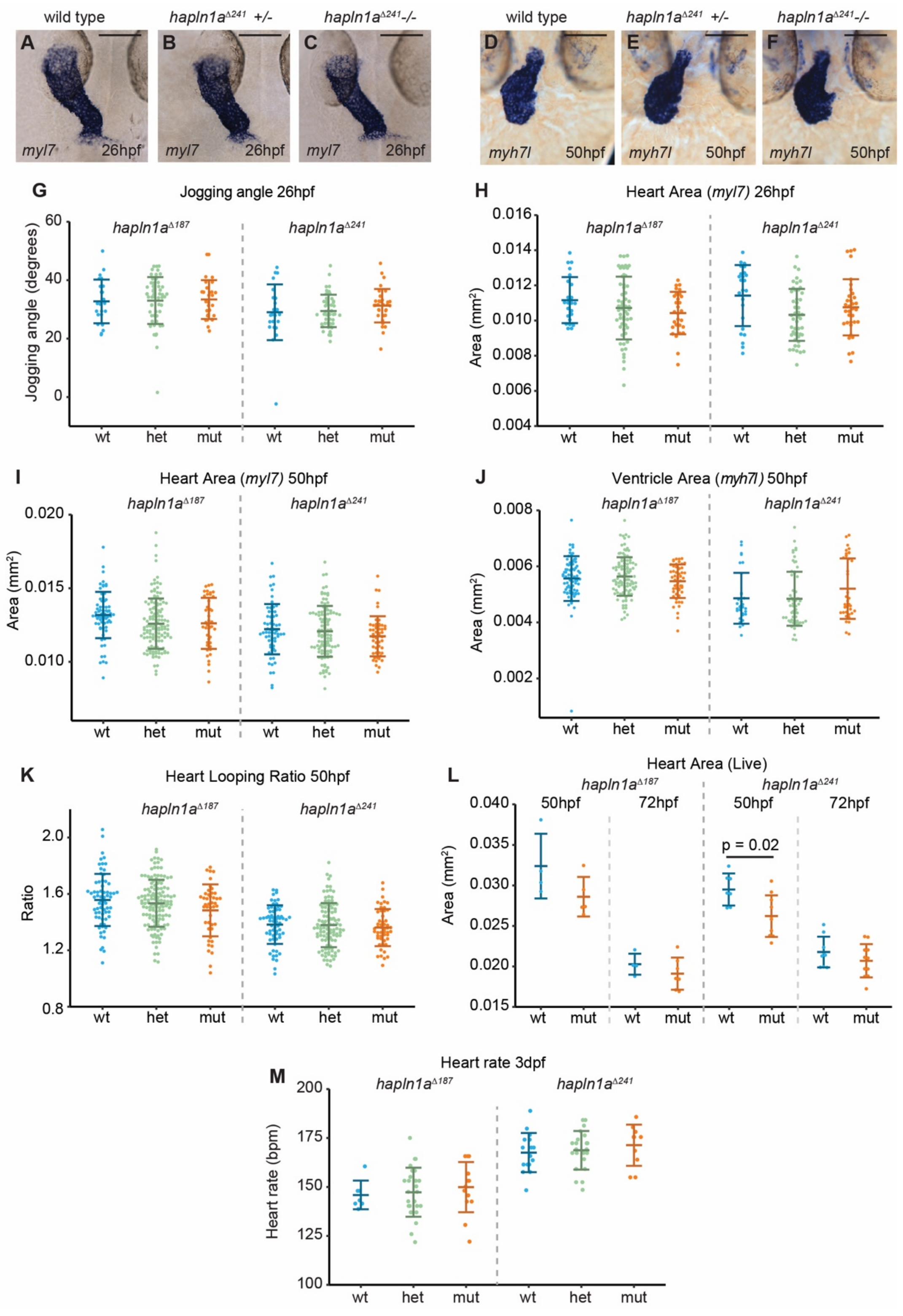

A-C: mRNA *in situ* hybridisation expression analysis at 26hpf of *myl7* in wild-type siblings (A), *hapln1a*$^{\Delta241}$ heterozygous siblings (B) or *hapln1a*$^{\Delta241}$ homozygous mutant embryos (C). D-F: mRNA *in situ* hybridisation expression analysis at 50hpf of *myh7l* in wild-type siblings

(D), *hapln1a*$^{\Delta241}$ heterozygous siblings (E) or *hapln1a*$^{\Delta241}$ homozygous mutant embryos (F). Scale bar = 50µm. G-H: Quantification of heart jogging angle (J) and heart size (K) from *myl7* expression at 26hpf in sibling and *hapln1a* mutant embryos. n = 23 *hapln1a*$^{\Delta187}$ wt; 56 *hapln1a*$^{\Delta187}$ het; 29 *hapln1a*$^{\Delta187}$ mut, 24 *hapln1a*$^{\Delta241}$ wt; 50 *hapln1a*$^{\Delta241}$ het; 34 *hapln1a*$^{\Delta241}$ mut. I: Quantification of heart size from *myl7* expression at 50hpf in sibling (wt/het) and *hapln1a* mutant embryos (mut). n = 69 *hapln1a*$^{\Delta187}$ wt; 133 *hapln1a*$^{\Delta187}$ het; 43 *hapln1a*$^{\Delta187}$ mut, 65 *hapln1a*$^{\Delta241}$ wt; 104 *hapln1a*$^{\Delta241}$ het; 51 *hapln1a*$^{\Delta241}$ mut. J: Quantification of ventricle size from *myh7l* expression at 50hpf in sibling (wt/het) and *hapln1a* mutant embryos (mut). n = 77 *hapln1a*$^{\Delta187}$ wt; 113 *hapln1a*$^{\Delta187}$ het; 43 *hapln1a*$^{\Delta187}$ mut, 32 *hapln1a*$^{\Delta241}$ wt; 60 *hapln1a*$^{\Delta241}$ het; 39 *hapln1a*$^{\Delta241}$ mut. K: Quantification of looping ratio from *myl7* expression at 50hpf in sibling (wt/het) and *hapln1a* mutant embryos (mut), number of embryos same as in (K). L: Quantification of heart size from live light-sheet images of sibling (wt) and *hapln1a* mutant embryos (mut) at 50hpf and 72hpf. n = 4 *hapln1a*$^{\Delta187}$ wt; 5 *hapln1a*$^{\Delta187}$ mut, 7 *hapln1a*$^{\Delta241}$ wt; 10 *hapln1a*$^{\Delta241}$ mut. Heart area is significantly reduced in *hapln1a*$^{\Delta241}$ mutants at 50hpf. M: Quantification of heart rate at 3dpf in sibling (wt/het) and *hapln1a* mutant embryos (mut). n = 7 *hapln1a*$^{\Delta187}$ wt; 26 *hapln1a*$^{\Delta187}$ het; 14 *hapln1a*$^{\Delta187}$ mut, 16 *hapln1a*$^{\Delta241}$ wt; 22 *hapln1a*$^{\Delta241}$ het; 10 *hapln1a*$^{\Delta241}$ mut. Mean ± SD is plotted. Comparative statistics carried out using a Kruskal-Wallis test with multiple comparisons.

Figure S6: *hapln1a* mutants have defects in atrial growth and morphology.

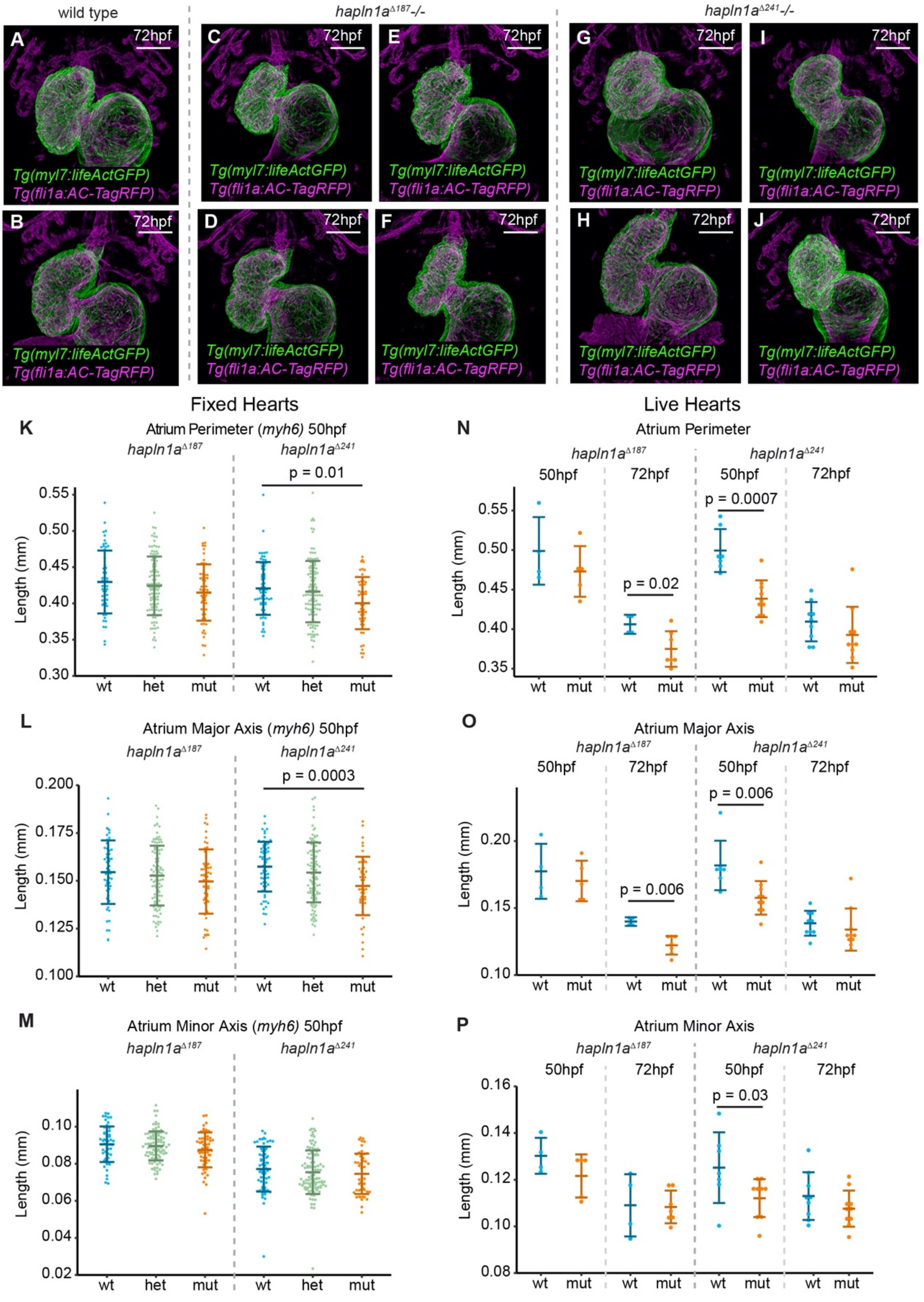

A-J: Maximum intensity projections of light-sheet z-stacks of live 72hpf *Tg(myl7:lifeActGFP); Tg(fli1a:AC-TagRFP)* transgenic wild-type (A, B), *hapln1a*$^{\Delta187}$ mutant embryos (C-F) and

hapln1a$^{\Delta241}$ mutant embryos (G-J). Scale bar = 50µm. K-M: Quantification of atrial perimeter (K) atrial major axis (L) and atrial minor axis (M) in *myh6*-stained ISH-processed sibling embryos (wt/het) and *hapln1a*$^{\Delta187}$ or *hapln1a*$^{\Delta241}$ mutants (mut) at 50hpf. Atrial perimeter and major axis is significantly reduced in *hapln1a*$^{\Delta241}$ mutants compared to wild type siblings. In K-M n = 54 *hapln1a*$^{\Delta187}$ wt; 104 *hapln1a*$^{\Delta187}$ het; 60 *hapln1a*$^{\Delta187}$ mut, 66 *hapln1a*$^{\Delta241}$ wt; 116 *hapln1a*$^{\Delta241}$ het; 53 *hapln1a*$^{\Delta241}$ mut. N-P: Quantification of atrial perimeter (N) atrial major axis (O), and atrial minor axis (P) in live light-sheet z-projections from sibling embryos (wt/het) and *hapln1a*$^{\Delta187}$ or *hapln1a*$^{\Delta241}$ mutants (mut) at 50hpf and 72hpf. Atrial perimeter, major axis and minor axis are significantly reduced in *hapln1a*$^{\Delta241}$ mutants compared to wild type siblings at 50hpf, and atrial perimeter and major axis are significantly reduced in *hapln1a*$^{\Delta187}$ mutants at 72hpf. In N-P n = 4 *hapln1a*$^{\Delta187}$ 50hpf wt; 5 *hapln1a*$^{\Delta187}$ 50hpf mut, 7 *hapln1a*$^{\Delta241}$ 50hpf wt; 10 *hapln1a*$^{\Delta241}$ 50hpf mut; 4 *hapln1a*$^{\Delta187}$ 72hpf wt; 7 *hapln1a*$^{\Delta187}$ 72hpf mut, 9 *hapln1a*$^{\Delta241}$ 72hpf wt; 10 *hapln1a*$^{\Delta241}$ 72hpf mut. Comparative statistics carried out using a Kruskal-Wallis test with multiple comparisons.

Figure S7: *hapln1a* morpholino is a loss-of-function model.

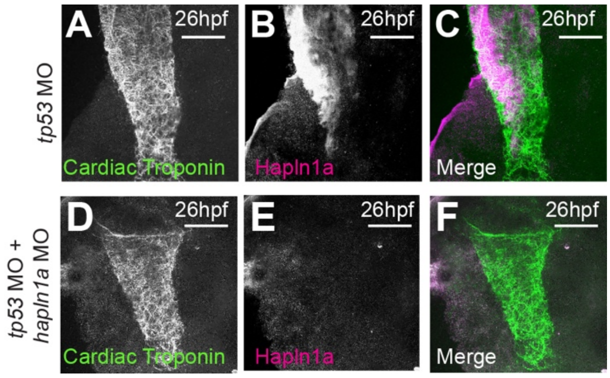

A-F: Immunohistochemistry analysis of Hapln1a (magenta) in embryos injected with either *tp53* MO (A-C) or *hapln1a* MO + *tp53* MO (D-F). Cardiac Troponin (green) outlines the heart. Hapln1a is present in *tp53* MO-injected embryos (n=5) but is absent in embryos injected with *hapln1a* MO + *tp53* MO (n=6). Scale bar = 50µm.

Figure S8: Regionalised ECM thickness is disrupted in *hapln1a* mutants

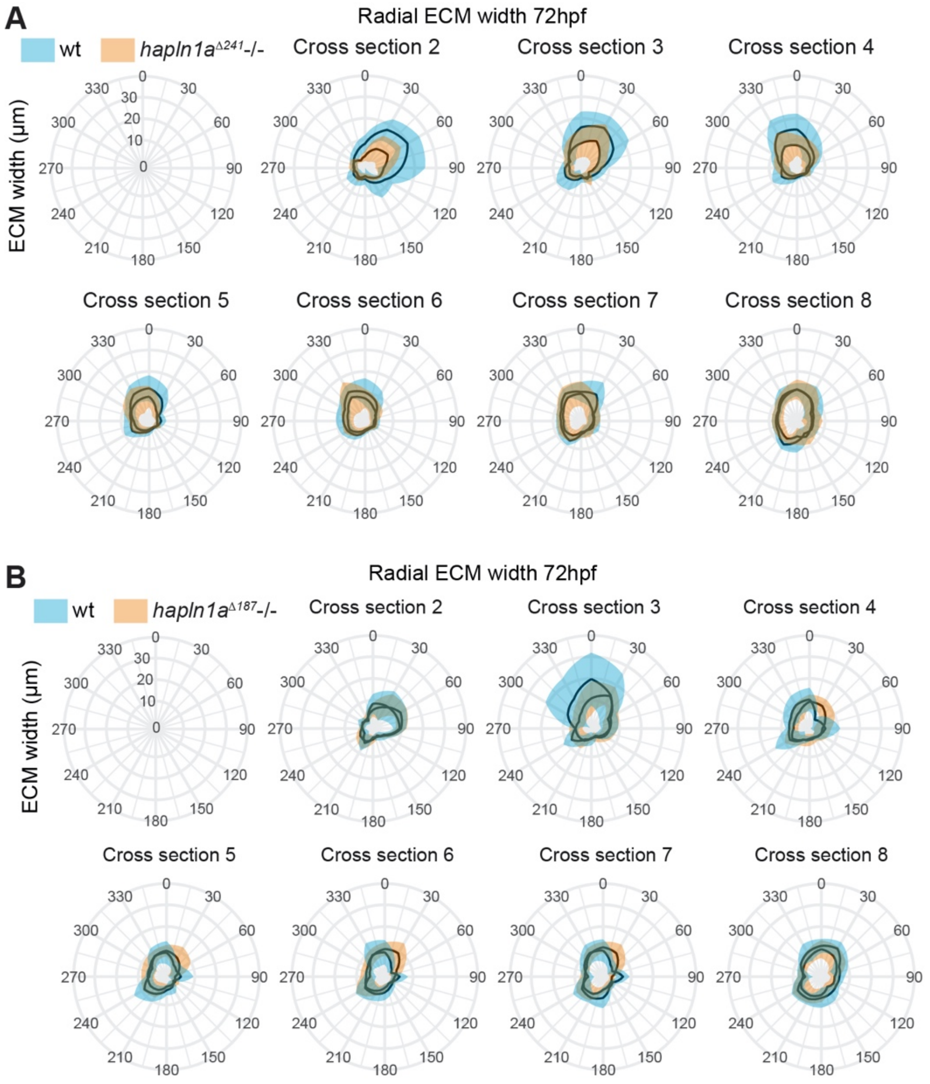

A-B: Quantification of ECM width of atrial cross sections at defined positions along the longitudinal axis of the atrium from AVC (cross-section 2) towards venous pole (cross-section 8) at 72hpf in *hapln1a*$^{\Delta 241}$ mutants (A) or *hapln1a*$^{\Delta 187}$ mutants (B). Wild type - blue, *hapln1a* mutant - orange. Mean ± SD are plotted. n ≥ 4 at each location.

Figure S9: HA is required at early stages of heart development for cardiac morphogenesis and interacts with Hapln1a

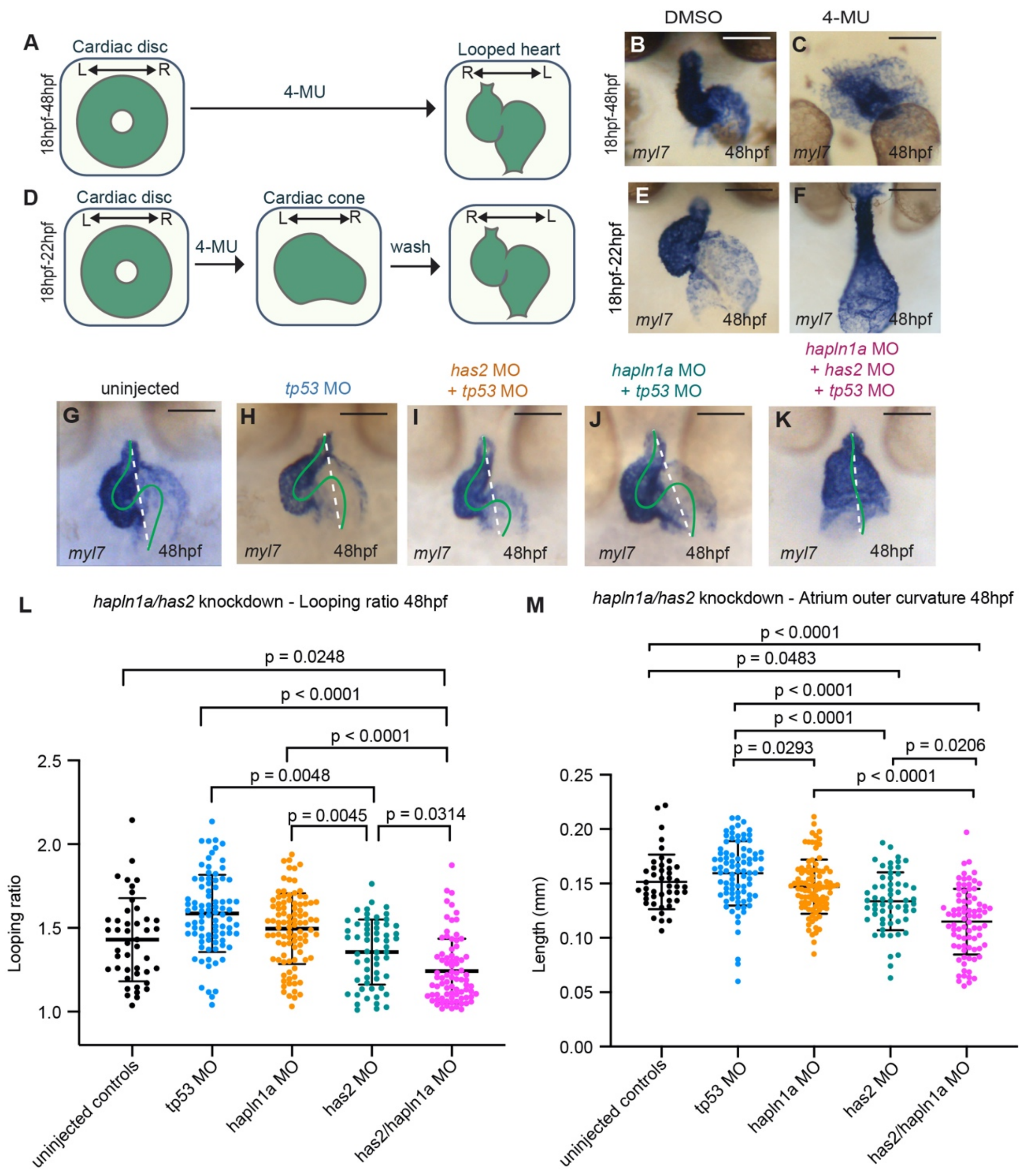

A, D: Schematic depicting timing of 4-MU treatments. B-C, E-F: *in situ* hybridisation analysis of *myl7* expression at 48hpf in embryos treated either with DMSO (B, E) or 4-MU (C, F). Embryos treated with 4-MU from 18hpf to 48hpf exhibit severe defects in heart development, characterised by a failure to correctly form the heart tube (C). In comparison, embryos treated with 4-MU in a shorter time window from 18hpf to 22hpf exhibit milder defects in heart

morphogenesis, characterised by a specific failure in heart looping morphogenesis (F). G-K: mRNA *in situ* hybridisation analysis at 48hpf of *myl7* expression in embryos injected with *tp53* MO (H), sub-phenotypic doses of either *has2* MO + *tp53* MO (I) or *hapln1a* MO + *tp53* MO (J), or a co-injection of sub-phenotypic doses of both *has2* and *hapln1a* MOs together with *tp53* MO (K). Embryos injected with sub-phenotypic doses of either *has2* MO + *tp53* MO or *hapln1a* MO + *tp53* MO do not exhibit heart defects, whereas embryos co-injected with both morpholinos together with *tp53* MO display unlooped hearts with dilated atria (K). Scale bar = 100µm. White dashed line = linear heart length, green line = looped heart length. L-M: Quantification of heart looping ratio (L) and atrial outer curvature (M) at 48hpf in embryos injected with *tp53* MO, sub-phenotypic doses of single *has2* or *hapln1a* MO, or co-injection of *has2* and *hapln1a* MOs. Embryos co-injected with *has2* and *hapln1a* MOs exhibit significantly reduced heart looping and atrial ballooning compared with single injected or uninjected controls. In L and M n = 44 uninjected controls; 85 *tp53* MO; 59 *tp53* + *hapln1a* MO; 95 *tp53* + *has2* MO; 81 *tp53* + *hapln1a* + *has2* MO. Median ± interquartile range are plotted, comparative statistics performed using Kruskal Wallis with multiple comparisons.

Movie S1: The cardiac ECM is asymmetric in the zebrafish heart tube

Live light-sheet optical cross-sections through the beating heart tube of a 26hpf *Tg(myl7:lifeActGFP); Tg(fli1a:AC-TagRFP); Tg(lft2BAC:Gal4FF); Tg(UAS:RFP)* at the level of the future atrium. The myocardium is marked in green and the dorsal myocardium and endocardium are marked in magenta. Movie speed is not in real-time.

Table S1: Number of embryos analysed for *hapln1a* morphant, *hapln1a* mutant and *pkd2* mutant experiments

<table>
<tr><th>Genotype</th><th>Stage/gene</th><th>Wild type</th><th>Heterozygous</th><th>Homozygous mutants</th></tr>
<tr><td>hapln1a$^{\Delta 187}$ mutants</td><td>26hpf/myl7</td><td>23</td><td>56</td><td>29</td></tr>
<tr><td></td><td>50hpf/myl7</td><td>69</td><td>133</td><td>43</td></tr>
<tr><td></td><td>50hpf/myh7l</td><td>77</td><td>113</td><td>54</td></tr>
<tr><td></td><td>50hpf/myh6</td><td>54</td><td>104</td><td>60</td></tr>
<tr><td></td><td>50hpf live</td><td>4</td><td>-</td><td>5</td></tr>
<tr><td></td><td>72hpf live</td><td>4</td><td>-</td><td>7</td></tr>
<tr><td>hapln1a$^{\Delta 241}$ mutants</td><td>26hpf/myl7</td><td>24</td><td>50</td><td>34</td></tr>
<tr><td></td><td>50hpf/myl7</td><td>65</td><td>104</td><td>51</td></tr>
<tr><td></td><td>50hpf/myh7l</td><td>32</td><td>60</td><td>39</td></tr>
<tr><td></td><td>50hpf/myh6</td><td>66</td><td>116</td><td>53</td></tr>
<tr><td></td><td>50hpf live</td><td>7</td><td>-</td><td>10</td></tr>
<tr><td></td><td>72hpf live</td><td>9</td><td>-</td><td>10</td></tr>
<tr><td>pkd2^{hu2173} mutants</td><td>19hpf/hapln1a</td><td colspan="2">72</td><td>13</td></tr>
<tr><td></td><td>26hpf/hapln1a</td><td colspan="2">138</td><td>40</td></tr>
<tr><td>spaw mutants</td><td>19hpf/hapln1a</td><td colspan="2">69</td><td>19</td></tr>
</table>

Experiment	Stage/ gene	Uninj.	*tp53* MO	*tp53* MO + *hapln1a* MO	*tp53* MO + *has2* MO	*tp53* MO + *hapln1a MO* +*has2* MO
hapln1a/	50hpf/	44	85	59	95	81

has2 MO	*myl7*				

Experiment	Stage/ gene	DMSO 18hpf – 48hpf	4MU 18hpf – 48hpf	DMSO 18hpf – 22hpf	4MU 18hpf – 22hpf
HA inhibition	48hpf/ *myl7*	38	36	36	44

Table S2: Raw Tomo-seq transcriptional analysis of Heart #1 at 26hpf

Heart 1 sectioned from venous pole to arterial pole, table includes Ensembl gene identifiers, raw read numbers per section, and ERCC spike in read numbers

Table S3: Spike-in normalised transcriptional analysis of Heart #1 at 26hpf

Heart 1 sectioned from venous pole to arterial pole, read numbers per section normalised to spike-in RNA, and including gene names.

Table S4: Raw Tomo-seq transcriptional analysis of Heart #2 at 26hpf

Heart 1 sectioned from arterial pole to venous pole, table includes Ensembl gene identifiers, raw read numbers per section, and ERCC spike in read numbers

Table S5: Spike-in normalised transcriptional analysis of Heart #2 at 26hpf

Heart 2 sectioned from venous pole to arterial pole, read numbers per section normalised to spike-in RNA, and including gene names.

Supplementary Methods

Generation of the Tg(lft2BAC:Gal4FF) transgenic line

The *Tg(lft2BAC:Gal4FF)* line was generated by recombineering of bacterial artificial chromosome (BAC) CH211-236P5 as previously described[1,2]. A Gal4FF_kan cassette was inserted at the ATG start codon of the first exon of the *lft2* gene. Amplification from a pCS2+Gal4FF_kanR plasmid was achieved with primers :

F_LFT2_GAL4FF

5'-

cctcagagcttcagtcagtcattcattctttcactggcatcgttagatcaACCATGAAGCTACTGTCTTCTATCGA

AC-3'

R_LFT2_NEO

5'-

tgtgtgagtgagatcgctgtggtcaaaatgaacagctggatgaacagagcTCAGAAGAACTCGTCAAGAAGGC

G-3'

Sequences homologous to the genomic locus in lower case. Recombineering was essentially carried out following the manufacturer's protocol (Red/ET recombination; Gene Bridges GmbH). BAC DNA isolation was carried out using a Midiprep kit (Life Technologies BV). BAC DNA was injected at a concentration of 300 ng/µl in the presence of 0.75U PI-SceI meganuclease (New England Biolabs) in 1-cell stage *Tg(UAS:GFP)* or *Tg(UAS:RFP)* embryos (both UAS lines[3]). At 1dpf, healthy embryos displaying robust *lft2*-specific fluorescence were selected and grown to adulthood. Founder fish (F0) were identified by outcrossing and the progeny (F1) was grown to establish the transgenic line.

Generation of $pkd2^{hu2173}$ allele

The $pkd2^{hu2173}$ allele was generated using ENU mutagenesis and consists of an A->T transversion at base position 1327 which results in a premature stop codon at amino acid 302 of 904. The truncation occurs in the first extracellular loop, before the channel pore, and is

predicted to be a null. The *pkd2*^{hu273} allele can be identified by PCR amplification with the following primers: forward primer 5'- GATTTATTGCTCTGTTTGTTTGTAAGGA-3' and reverse primer 5' -GAAGTCCAAGAACACCGCTC-3', followed by XmnI restriction of the PCR product. The primers contain a mismatch which together with the *pkd2*^{hu2173} mutation introduces an XmnI recognition site into the mutant strand.

in situ hybridisation mRNA probes

The following primers were used to amplify a region of the CDS from the gene of interest:

Gene	Forward Primer	Reverse Primer	Size (bp)
hapln1a	5' – TGGCATTGATGGTGTTTGCA - 3'	5' – ACAGTTCCGTCACTAAGCCA - 3'	860
has2	5' – GTTCACGCAGACCTCATCAC – 3'	5' – CATCCAATACCTCACGCTGC – 3'	1050
acana	5'- CGGATCAAGTGGAGTCTGGT -3'	5'- GAAGGGAGGACGTGGGAAAT -3'	1067
acanb	5'- ATCAAGACAGCACCCTCAGT -3'	5'- TTTCTGGAAATGGCGTGGTC -3'	1035
chsy1	5'- CACCATTCAGCTCCATCGTG-3'	5'- TCGGCTTTGGGGTACTTCAT-3'	801

All probe sequences were ligated into the PCR2-TOPO vector (Invitrogen). *myl7*, *fli1a*, *vcana* and *vcanb* mRNA probes have been previously described[4–6].

Pharmacological treatments

To block HA synthesis, 4-Methylumbelliferone (4-MU, Sigma-Aldrich) was dissolved in DMSO to a stock concentration of 100mM, and subsequently diluted to a working concentration of 1mM in E3 medium. Embryos were dechorionated and incubated in 4-MU or an equal concentration of DMSO (1%). For timed treatments, at the end of the treatment window embryos were washed 3 x 5 mins in E3 to remove the 4MU, before being placed in fresh E3 until fixation.

Heart rate analysis

Embryos were transferred individually from a 28.5°C incubator and positioned laterally on an agarose mold (2% agarose in E3 medium) for imaging. Once the heart was in focus under the stereoscope (using 5x magnification), image sequences of 5000ms in duration were acquired at 120 frames per second, using a High Speed Camera (Chameleon3 USB3, FLIR Integrated Imaging Solutions Inc) together with SpinView software (Spinnaker v. 2.0.0.147). Image sequences were imported to Fiji[7] and heart rate obtained through a custom-written macro incorporating the MultiKymograph plugin to obtain a time-space plot that captured the motion of the heart, and Plot Profile plugin to measure the time between heart beats. Individual points plotted in graphs show average heart rate for each embryo over the 5000ms imaging window.

Image quantification

Light-sheet images of heart morphology: Image stacks were initially processed using Vision4D (Arivis AG, Germany) and Fiji. Processing steps included noise removal, background correction, and subsequent application of individual morphological filters to each channel to sharpen the edges of the myocardial and endocardial tissue layers. Maximum intensity z-projections of the composite channels were used to visualise cardiac morphology.

Analysis of ECM width in *ssNcan-GFP* mRNA injected embryos: Images were acquired on an Airyscan microscope with a z-resolution of 1µm step size. Images were Airyscan processed using Zen Black software (Zeiss), and the resulting image stacks were optically resliced using Fiji. ECM width was manually measured in Fiji. ECM measurements were aligned between samples at the venous pole of the heart for plotting.

Analysis of radial ECM width in live embryos: Maximum intensity projections of 50hpf and 72hpf light-sheet images of live *Tg(myl7:lifeActGFP); Tg(fli1a:AC-TagRFP)* transgenic hearts were used to draw the outer and inner curvatures of the atrium. Using a custom-written ImageJ

macro these two splines were then divided into segments of equal length by 12 equidistant points. Corresponding points from each of the two curvature splines were subsequently connected to define the cross-section positions starting from the AVC towards the venous pole (Figure 4E). Cross-section slices at the inflow tract and atrioventricular canal where the atrial signal is not complete were removed from the analysis. All cross sections were blinded, and a radial grid with 30° gridlines placed on each image, manually centred at the middle of the chamber lumen, where 0° represents the dorsal face and 180° the ventral face of the heart. The width of the ECM was manually measured at each position on the radial grid where both myocardial and endocardial layers were visible. Where myocardium and endocardium were touching, ECM width was set to 1px (0.2μm). To visualise radial ECM spread, the average ECM width and deviation at each radial position was calculated for each slice.

Looping morphology: Looping ratio was calculated from images of *myl7* expression detected by ISH. All samples from one experimental set were blinded using the ImageJ Blind_Analysis plugin (https://github.com/quantixed/imagej-macros/blob/master/Blind_Analysis.ijm). The linear distance from arterial to venous poles of the heart was measured as a straight-line distance, and looped distance was drawn from the same positions at each pole through the centre of each chamber, down the centreline of the looped heart. Looping ratio was determined by dividing looped distance by the linear distance. Statistical testing of average looping ratio between experimental conditions was carried out using Kruskal-Wallis with Dunn's multiple comparisons.

Heart jogging: The angle of heart jogging was calculated from images of *myl7* expression detected by ISH. A line was drawn down the midline of the embryo, and a second line drawn through the midline of the heart tube, using the centre of the inflow and outflow tracts to standardise line positioning. The angle of intersection between these two lines defines heart jogging angle.

Heart and chamber parameters: whole-heart, ventricle or atrium parameters at 26hpf and 50hpf were quantified from *in situ* hybridisations by manually drawing round either *myl7*, *myh7l* or *myh6* staining area in Fiji. Heart and atrial parameters in live embryos at 50hpf and 72hpf were quantified from maximum intensity projections of z-stacks obtained on the light-sheet by manually drawing round the heart or atrium using the *Tg(myl7:lifeActGFP)* channel. Atrial curvatures were quantified by manually drawing along the atrium from the AVC to the inflow tract of the heart. Atrial major and minor axes quantifications were derived from an ellipse fit to the outline of the atrium. In embryos where heart looping morphology is profoundly affected, the atrioventricular boundary is defined using a combination of mild changes in tissue morphology (from concave to convex indicating transition from an area of tissue restriction (canal) to expansion (chamber)) together with intensity of *myl7 in situ* hybridisation staining, which is more intense in ventricular tissue when compared to atrial tissue.

Statistical testing of heart or chamber parameters between genotypes was carried out using Kruskal-Wallis with Dunn's multiple comparisons.

For examples of the quantifications of cardiac and chamber parameters described above, please refer to Figure S4.

hapln1a overexpression: Quantification of *hapln1a* overexpression was performed by imaging overexpression embryos where *myl7* expression was detected using INT/BCIP and *hapln1a* expression detected using NBT/BCIP. All embryos were imaged using the same microscope settings, and individual images combined into a composite of all experiments. Using the composite, channels were split in Fiji, resulting in the blue and green channels carrying the *myl7* stain, and the red channel carrying the *hapln1a* stain. Background was subtracted in the red channel. The *myl7* staining was used to calculate looping ratio for each heart. In addition, each *myl7* signal was used to manually trace the whole heart, atrium, AVC and ventricle for each heart, which was saved as a region of interest (ROI) and the area of each chamber was

measured in pixels. Next, the *hapln1a* image was thresholded to generate a binary image. The whole-heart, or chamber-specific ROI was applied to the thresholded *hapln1a* channel, and the number of positive pixels in each ROI recorded. Number of positive pixels as a percentage (%) of the total number of pixels comprising the heart or the specific chamber was calculated and plotted against looping ratio for each heart. Spearmann's correlation coefficient (r) was calculated in GraphPad Prism, with 95% confidence intervals.

Data was visualised using the ggplot2 package in R, or using GraphPad Prism.

References

1. Bussmann J, Schulte-Merker S. Rapid BAC selection for tol2-mediated transgenesis in zebrafish. *Development* 2011;**138**:4327–4332.

2. Tessadori F, Weerd JH van, Burkhard SB, Verkerk AO, Pater E de, Boukens BJ, Vink A, Christoffels VM, Bakkers J. Identification and Functional Characterization of Cardiac Pacemaker Cells in Zebrafish. *PLoS One* 2012;**7**:e47644.

3. Asakawa K, Kawakami K. Targeted gene expression by the Gal4-UAS system in zebrafish. *Dev Growth Differ* 2008;**50**:391–399.

4. Yelon D, Horne SA, Stainier DYR. Restricted Expression of Cardiac Myosin Genes Reveals Regulated Aspects of Heart Tube Assembly in Zebrafish. *Dev Biol* 1999;**214**:23–37.

5. Kang JS, Oohashi T, Kawakami Y, Bekku Y, Belmonte JCI, Ninomiya Y. Characterization of dermacan, a novel zebrafish lectican gene, expressed in dermal bones. *Mech Dev* 2004;**121**:301–312.

6. Brown LA, Rodaway AR, Schilling TF, Jowett T, Ingham PW, Patient RK, Sharrocks AD. Insights into early vasculogenesis revealed by expression of the ETS-domain transcription factor Fli-1 in wild-type and mutant zebrafish embryos. *Mech Dev* 2000;**90**:237–252.

7. Schindelin J, Arganda-Carreras I, Frise E, Kaynig V, Longair M, Pietzsch T, Preibisch S, Rueden C, Saalfeld S, Schmid B, Tinevez J-Y, White DJ, Hartenstein V, Eliceiri K, Tomancak P, Cardona A. Fiji: an open-source platform for biological-image analysis. *Nat Methods* 2012;**9**:676–682.
